# Identifying viral infections through metagenomic Next Generation Sequencing of undiagnosed respiratory fevers in Madagascar (2014–2019)

**DOI:** 10.1186/s12879-026-13715-7

**Published:** 2026-06-06

**Authors:** Sophie Lockwood, Hafaliana Christian Ranaivoson, Tsiry Hasina Randriambolamanantsoa, Norosoa Razanajatovo, Vololoniaina Raharinosy, Vida Ahyong, Jean-Michel Héraud, Philippe Dussart, Vincent Lacoste, Cara E. Brook

**Affiliations:** 1 Department of Integrative Biology, University of California Berkeley, Berkeley, CA USA; 2 Department of Ecology and Evolution, University of Chicago, Chicago, IL USA; 3 Virology Unit, Institut Pasteur de Madagascar, Antananarivo, Madagascar; 4 Chan Zuckerberg Biohub, San Francisco, CA USA

**Keywords:** Respiratory viruses, mNGS, Public health surveillance

## Abstract

**Background:**

Respiratory illness contributes to substantial global morbidity and mortality. In Madagascar, an island nation off the southeastern coast of the African continent, hospital-based public health surveillance for respiratory pathogens screens for common respiratory viruses. However, many cases remain undiagnosed.

**Methods:**

We conducted metagenomic Next Generation Sequencing (mNGS) to identify the pathogen profile of 102 undiagnosed febrile patients who presented to public hospitals with respiratory symptoms and screened negative on a 14-virus multiplex RT-qPCR. We analyzed the diversity of the respiratory microbiome of each patient from mNGS data and identified viral infections potentially linked to undiagnosed fever. We assembled whole genome consensus sequences of viruses with sufficient read depth and coverage, characterized each phylogenetically, and identified any discrepancies with the primers used in the multiplex RT-qPCR panel. Finally, we compared all whole genome sequences against publicly available global databases in a phylogenetic analysis.

**Results:**

We identified evidence of infection by a wide range of known human viruses in approximately two thirds (64.7%) of study participants from nine different families of viruses and generated 30 complete or nearly complete consensus sequences of known respiratory viruses including orthopneumoviruses, metapneumoviruses, rhinoviruses, coronaviruses, parainfluenza virus, and bocaparvovirus. mNGS-attributed evidence of infection was predominantly due to orthopneumovirus (also called respiratory syncytial virus [RSV]; *n* = 24; *n* = 8 previously diagnosed) and rhinovirus (*n* = 18) detections, despite previous negative RT-qPCR results for the majority of these cases. Finally, phylogenetic analysis identified two distinct phylogenetic clusters of RSV subtype A, suggesting local transmission following distinct international introductions for this virus.

**Conclusion:**

mNGS provides a sensitive pan-pathogenic tool for virus detection. We demonstrate the diversity of viruses associated with undiagnosed respiratory fevers in Madagascar, emphasize the importance and relevance of the existing respiratory surveillance in the country, and highlight the interconnectedness of regional respiratory infection dynamics with global networks of respiratory pathogen transmission.

**Supplementary Information:**

The online version contains supplementary material available at 10.1186/s12879-026-13715-7.

## Background

Respiratory illness is responsible for substantial global morbidity and mortality. A leading cause of death due to infectious disease, respiratory infections result in the highest burden in lower- and middle-income countries and among children under age five [1–3]. Viruses frequently cause acute respiratory infections, although bacterial and parasitic infections can also result in respiratory symptoms [2, 4].

Madagascar, an island nation of nearly 33 million people off the southeastern coast of the African continent, experiences a high burden of respiratory disease [5], resulting from a high diversity of pathogens [6]. In Madagascar, viral infections contribute to approximately one fifth of hospital visits for non-specific febrile disease [6] and more than half of hospital visits for respiratory symptoms in both urban [7] and rural [8] settings. Madagascar is a relatively closed island population with temperate to tropical climates where respiratory virus dynamics likely reflect both local circulation and repeated introductions from outside sources. Respiratory pathogen surveillance in Madagascar has permitted the identification of temporal [7], climatic [9] and geographic [10] patterns of acute respiratory viral infections. Interestingly, the seasonal patterns observed in influenza and parainfluenza transmission in Madagascar are asynchronous with global patterns, even those of countries connected by relatively frequent travel [11, 12]. Further, Madagascar hosts unique viruses relative to global trends, with distinct circulating strains of influenza virus in comparison to outside strains used to determine annual vaccine recommendations [13]. Madagascar’s unusual geographic position and infection seasonality underscore the importance of investigating patterns in respiratory illness in the country.

The Ministry of Health of Madagascar, in collaboration with the Institut Pasteur de Madagascar (IPM), established a nationwide sentinel syndromic surveillance system for febrile illness in 2007, expanding coverage from surveillance that had previously only been conducted in the capital and largest city, Antananarivo [14]. The system included screening for influenza-like illnesses via nasopharyngeal sampling and provided a useful tool for monitoring abnormal febrile illness and tracking respiratory pathogen outbreaks (e.g. the influenza A/(H1N1)pdm09 and SARS-CoV-2 viruses) [15]. Select hospitals in the country screen patients reporting with respiratory symptoms and fever for influenza viruses, respiratory syncytial virus (RSV), and, since 2020, SARS-CoV-2 by testing nasopharyngeal swabs with real-time quantitative polymerase chain reaction (RT-qPCR). During outbreak investigations, patients are screened for a larger suite of common pathogens with a 14-virus multiplex RT-qPCR panel that includes strains of parainfluenza viruses, coronaviruses, rhinoviruses, influenza viruses, adenoviruses, and bocaviruses [7]. However, 25% of patients reporting to Malagasy health centers with influenza-like illness between 2008 and 2009 [7] and 80% of symptomatic SARS-CoV-2 negative patients in hospitals and communities between 2020 and 2022 [16] remained undiagnosed after RT-qPCR screening for these 14 viruses.

Metagenomic Next Generation Sequencing (mNGS) enables identification of pathogens of unknown origin, by sequencing all nucleic acid in a single sample, allowing for *de novo* genome assembly of both novel and previously described microbes of viral, bacterial, fungal, or eukaryotic origin that may be associated with febrile illness. As an unbiased method, mNGS is an effective surveillance tool for non-specific conditions such as febrile disease with respiratory symptoms. The utility of mNGS in identifying etiologic agents of disease has been previously demonstrated in studies of febrile illness in low-resource settings in Cambodia [17] and Uganda [18, 19], to investigate the etiology of undiagnosed dengue-like symptoms in Nicaragua [20], and to provide diagnosis of fever of unknown cause in travelers to the United Kingdom [21] and the Netherlands [22]. As a tool, mNGS has led to the identification of novel human viruses [23, 24], including those of zoonotic origin [25]. Molecular epidemiology played a critical role in the COVID-19 pandemic, and led to a global expansion of sequencing capacity, although regional disparities in sequence reporting persist [26]. In low and middle income countries (LMICs), mNGS platforms for pathogen surveillance offer unique benefits and face additional hurdles [26, 27]. A pan-pathogenic detection tool, mNGS is useful when samples are limited in quantity and has the potential to enhance biological surveillance in regions where cross-species transmission risk is high. However, establishing the laboratory and bioinformatics pipeline necessary to carry out mNGS is an ongoing challenge as it requires advanced training, costly equipment and reagents, and reliable cold-chain, electricity, and internet infrastructure.

Here, we describe five years of sampling with subsequent mNGS sequencing and analysis of undiagnosed febrile patients reporting with respiratory symptoms to public hospitals primarily in Antananarivo, Madagascar. We find evidence of viral infection in nearly two thirds of cases and generate consensus genomes of respiratory viruses known to circulate in Madagascar, including human metapneumoviruses, rhinoviruses, seasonal coronaviruses, and orthopneumoviruses (RSV). We report factors associated with viral detection, contextualize the identified viruses, and assess primer compatibility between the RT-qPCR surveillance panel and the sequences. Finally, we conduct phylogeographic analyses to estimate the number of introductions of RSV subtype A, the only pathogen detected in this study with sufficient frequency to support a broader analysis.

## Methods

### Study population and sample collection

Patients admitted to public hospitals in Madagascar with fever between 2014 and 2019 were screened according to syndromic surveillance guidelines. Nasopharyngeal swabs were collected from febrile patients with cough and were transported to the IPM laboratory in Antananarivo where they underwent RNA extraction and screening by real-time RT-PCR for 14 routine human respiratory viruses: human parainfluenza viruses 1, 2, and 3, human coronaviruses OC43, 229E, HKU1, and NL63, human rhinovirus (HRV), alphainfluenza and betainfluenza viruses, human metapneumovirus, RSV, human adenovirus, and human bocavirus (see Table S1 for details). Nasopharyngeal samples analyzed in this study screened negative on this pathogen panel and later underwent mNGS. Eleven nasopharyngeal swabs from individuals who screened positive for RSV, HRV, and influenzavirus were included as positive controls, representing only a subset of the total number of febrile patients who screened positive during the study interval. Additionally, two healthy individuals provided nasopharyngeal samples for negative controls.

### mNGS sample processing, sequencing, and bioinformatics

Following sample collection, RNA extraction was conducted using the Zymo Quick DNA/RNA Microprep Plus Kit (Zymo Research, Irvine, CA) according to the manufacturer’s instructions and including the reaction for DNAse digestion. mNGS library preparation was completed using the NEBNext Ultra RNA II Library Prep Kit (New England BioLabs, Beverly, MA, USA) also per manufacturer instructions. Libraries were pooled and assessed for quality with electrophoresis (High-Sensitivity DNA Kit and Agilent Bioanalyzer; Agilent Technologies, Santa Clara, CA, USA) and small-scale sequencing (2 × 146 bp) on an Illumina iSeq machine (Illumina, San Diego, CA, USA) to permit equimolar pooling of individual libraries that proceeded to large-scale paired end mNGS (2 × 146 bp) on an Illumina NextSeq sequencing machine (Illumina, San Diego, CA, USA). Using the Chan Zuckerburg Infectious Disease (CZID) bioinformatics pipeline v8.3 [28], raw sequence reads were progressively filtered for host reads, read quality, and duplicates (see Figure S1). In brief, adapter trimming and removal of short and low complexity reads was conducted with CZID’s customized version of fastp [29], which removes bases with low quality scores, reads shorter than 35 base pairs, reads containing more than 15 undetermined bases, and reads comprising more than 40% low complexity regions. Host (human) reads were identified and removed using Bowtie 2 [30] and HISAT2 [31]. Removal of duplicates was based on identity in the first 70 bp and was conducted using CZID’s dedup program. *De novo* genome construction was conducted on the resulting filtered non-host reads; read alignment to prior sequences was assessed via both NCBI nucleotide (NT) and amino acid (NR) databases using adaptions of Minimap2 [32] and DIAMOND [33]. Contig assembly was performed using SPAdes [34] and Bowtie 2 [30]. Microbial taxa were identified based on BLAST nucleotide and protein comparison to NCBI databases [35].

Where possible, consensus genomes were generated using the CZID pipeline, which aligns filtered non-human reads to a provided reference genome, which we specified as the closest identified sequence from BLASTN comparison, using Minimap2 [32]. Aligned reads were trimmed using Trim Galore (https://www.bioinformatics.babraham.ac.uk/projects/trim_galore/), which removes adapter sequences, low quality reads, and reads shorter than 20 bp. Consensus genomes were constructed using the iVar consensus tool [36], which calls bases with depths of 10 or more reads and assigns an N when sufficient bases cannot be called. After running the CZID consensus genome pipeline, we evaluated resulting genomes manually in Geneious Prime (2024.0.5) by realigning all reads to the CZID-generated consensus genome and verifying unspecified bases. Complete and nearly complete consensus sequences (*n* = 30) were added to GenBank (accession numbers: PX467117-PX467146), and raw data were uploaded to the NCBI Sequence Read Archive (SRA) under BioProject number PRJNA1328692.

### mNGS analysis

We sought to identify viruses associated with febrile disease. We followed previous methods [17] to consider identified viruses to be significant infections associated with febrile disease if reads generated from mNGS produced a matched alignment of at least 50 base pairs in length, with a depth of at least 10 reads per million, and an e-value of 1e-10 or lower based on BLAST comparison to the NT database. We implemented a background model in the CZID pipeline [28] composed of the two healthy human controls and generated z-score values comparing presence in reads per million of taxa in the samples and the background controls (see Supplement Text S1 for details). We further required that reads mapping to target taxa generated a z-score of at least 1 following comparison with this background microbiome. Both e-values and z-scores were generated using the CZID pipeline [28].

### Simple statistics

We analyzed patient demographic characteristics and compared characteristics between samples with and without evidence of viral infection using Chi squared and Fisher’s exact tests for categorical variables and the Wilcoxon rank-sum test for differences in age. We conducted simple linear regression to evaluate association between the number of patients with and without evidence of viral infection sampled in each month during the study interval. All analyses were conducted using R version 4.4.1.

### Microbial diversity

We assessed microbial diversity for each sample by calculating Shannon diversity indices for each sample in R version 4.4.1 using the Vegan package (https://vegandevs.github.io/vegan/). To calculate Shannon diversity, we used the number of different microbial species in each sample as called by Minimap2 [32] and DIAMOND [33] in the CZID pipeline [28] as the measure of richness and the number of reads assigned to each species as the measure of evenness. Here, we did not impose our alignment length, depth, or z- or e-score requirements to generate these data; all taxa in all samples were considered under the same base CZID identification parameters to generate analyses of microbial diversity. We compared samples with and without evidence of viral infection as well as to samples from two healthy controls. Differences in the mean Shannon diversity indices between groups were evaluated by ANOVA.

### Analysis of primer match

To identify compatibility between sequences assembled from Malagasy patients to the primer set included in the 14-pathogen surveillance screening, we downloaded all primer sequences (described in previously published work [7]). We aligned consensus genomes from this study with these primer sequences using GeneiousPrime and calculated percent pairwise identity and number of divergent base pairs between the primer set and each consensus genome corresponding to a pathogen included in the surveillance panel.

### Sequence analysis

We first conducted manual BLASTN comparison of each consensus genome with the NCBI database. Then, we conducted two main phylogenetic analyses: (1) full-genome maximum likelihood phylogenies for the family or genus of the viruses for which consensus genomes were obtained and (2) characterization of potential sources of introduction of RSV subtype A lineages, the only pathogen identified in our data with sufficient frequency for such an approach.

In the first phylogenetic analysis, we aligned our assembled consensus sequences with all sequences available in the RefSeq database [37] on NCBI Virus corresponding to the same viral family, sub-family or genus of the target sequences of interest. As such, we combined our two Madagascar *Coronaviridae* sequences with 74 reference sequences (Taxid: 11118) and the reference sequence for porcine torovirus (accession number: NC_022787.1) as an outgroup. We combined the sole Madagascar *Parvovirinae* sequence with 48 reference sequences (Taxid: 40119) and the reference sequence for Danaus plexippus iteravirus (accession number: NC_023842.1) as an outgroup. To make the *Paramyxoviridae* dataset, we combined the unique Madagascar sequence with 92 reference sequences (Taxid: 11158) and included the reference sequence for Sun Coast virus (accession number: NC_025345.1) as an outgroup. We combined 15 Madagascar *Pneumoviridae* sequences with 11 reference sequences (Taxid: 11244) and the Sun Coast virus (accession number: NC_025345.1) as an outgroup. We finally combined 11 Madagascar *Enterovirus* sequences with 31 reference sequences (Taxid: 12059) and the reference sequence for porcine sapelovirus (accession number NC_003987.1) as an outgroup. Accession numbers for all reference sequences included in all phylogenies are listed in Table S2.

Sequences from each of these datasets were aligned using default parameters in the online software MAFFT v7.511 [38] and visually verified in AliView [39]. Alignments were tested for the best fit nucleotide substitution model using ModelTest-NG [40]. Phylogenetic trees were inferred by maximum likelihood using the best-fitting model from ModelTest-NG, in all cases a general time reversible model with gamma distributed rates and invariant sites (GTR+I+G4), in RAxML [41]. We conducted Felsenstein’s [42] bootstrapping using RAxML and applied the MRE-based bootstopping test to evaluate diagnostic statistics after every 50 replicates [43]. Bootstrapping was terminated once diagnostic statistics fell below the threshold values, and support values were drawn on the best-scoring tree.

To contextualize RSV subtype A lineages in Madagascar with concurrently circulating global strains, we generated a comprehensive sequence dataset by compiling all the RSV-A consensus genomes obtained from this study with all publicly available complete and near-complete RSV-A genomes with available collection location and date corresponding to anytime during or before the same sampling interval (2014–2019) from NCBI Virus and GISAID [44] databases. The reference sequence of RSV subtype B (accession number NC_001781.1) was included as an outgroup. We then subsampled the dataset to a maximum four sequences per country per month during the interval of interest using a Nextstrain pipeline [45]. Model selection and phylogenetic tree reconstruction were both conducted using IQ-TREE 2.0.7 [46], and bootstraps were generated using ultrafast approximation [47] with 1,000 replicates.

All tree visualizations were conducted using ggtree [48] in R version 4.4.1.

## Results

### Clinical, demographic, and geographic characteristics

**Fig. 1:**
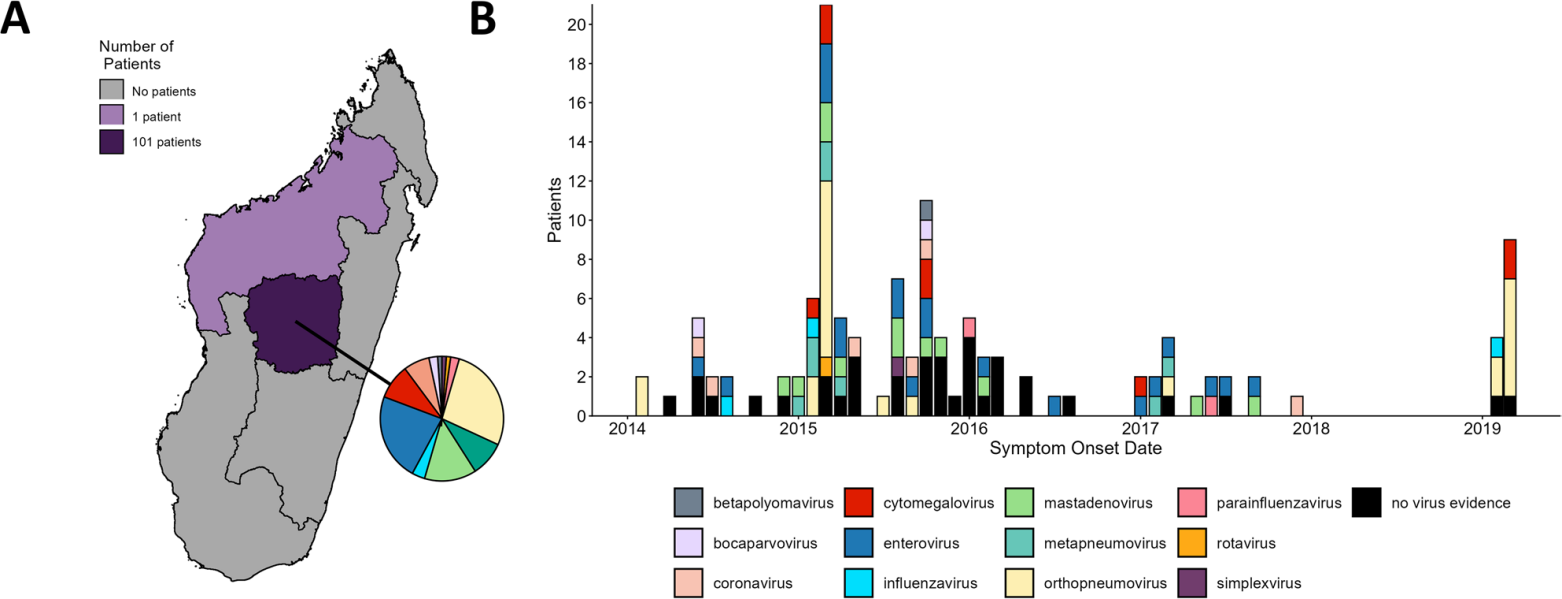
**A**) Province-level map of Madagascar displaying locations where patient samples were collected. Background color corresponds to the number of individuals sampled in each location. Pie charts indicate the type of viruses identified in the patient population of the three geographic regions in which patient samples yielded evidence of viral infection. Provinces where no samples had evidence of viral infection do not have pie charts. **B**) Time series of all nasopharyngeal sample collections between 2014 and 2019, with samples that yielded evidence of viral infection indicated in color. Each bar corresponds to one month of sampling

We analyzed 102 nasopharyngeal swabs collected from febrile patients in Antananarivo and Mahajanga provinces across Madagascar between 2014 and 2019 (Table 1), 11 of which were previously diagnosed samples by RT-qPCR included as positive controls. Two additional samples were collected from healthy controls in 2019. Febrile patients ranged in age from infants to adults but skewed towards pediatric patients (median age: 2 years, IQR: 0–29.75 years). When excluding positive controls, undiagnosed febrile patients were less skewed towards younger patients (median age: 4 years, IQR: 0–33.5). The median patient age was younger among individuals with evidence of viral infection (with viral infection: 1, IQR: 0–5.8 years; without viral infection: 31 years, IQR: 4.5–55.8 years, Wilcoxon rank-sum, *p* < 0.001), a pattern that held when the analysis was repeated excluding RSV infections (Wilcoxon rank-sum, *p* < 0.001). When considering just PCR-negative participants (e.g. excluding positive controls), this trend still held (with viral infection: 1, IQR: 0–19; without viral infection: 32.5, IQR: 6.25, 57.25). Both males and females were included in the sample (63% male), and sex did not differ between virus-infected and uninfected patients (χ^2^, *p* = 1). Most patients providing nasopharyngeal swabs presented to hospitals in Antananarivo, the central province that includes the capital city; only one sample was provided from Mahajanga province (Fig. 1).

**Table 1:** Febrile participant demographic characteristics stratified by evidence of viral infection, including both the 91 patients undiagnosed at time of hospital visit and the 11 patients previously diagnosed by RT-qPCR. Evidence of viral infection was determined from analysis of the mNGS reads from nasopharyngeal samples of patients reporting with fever and respiratory symptoms to hospitals across Madagascar; a read depth of at least 10 reads per million and statistical support for pathogen identification were considered evidence of viral infection

	Total* (N* = 102)	Evidence of viral infection (*N* = 66)	No evidence of viral infection (*N* = 36)
Age (years)	N (%)	N (%)	N (%)
0–5	59 (57.8)	49 (74.2)	10 (27.8)
6–9	3 (2.9)	2 (3.0)	1 (2.8)
10–19	4 (3.9)	1 (1.5)	3 (8.3)
20–29	10 (9.8)	7 (10.6)	3 (8.3)
30–39	6 (5.8)	1 (1.5)	5 (13.9)
40–49	6 (5.8)	2 (3.0)	4 (11.1)
50–59	5 (4.9)	2 (3.0)	3 (8.3)
60+	9 (8.8)	2 (3.0)	7 (19.4)
Sex			
Male	64 (62.7)	41 (62.1)	23 (63.9)
Female	38 (37.3)	25 (37.8)	13 (36.1)
Location			
Antananarivo	101 (99.0)	66 (100)	35 (97.2)
Mahajanga	1 (0.9)	0 (0)	1 (2.8)
Year of Sampling			
2014	14 (13.7)	8 (12.1)	6 (16.7)
2015	48 (47.1)	33 (50.0)	15 (41.7)
2016	14 (13.7)	3 (4.5)	11 (30.6)
2017	15 (14.7)	13 (19.7)	2 (5.6)
2018	0 (0)	0 (0)	0 (0)
2019	11 (10.8)	9 (13.6)	2 (5.6)

### Microbes detected by mNGS

**Fig. 2:**
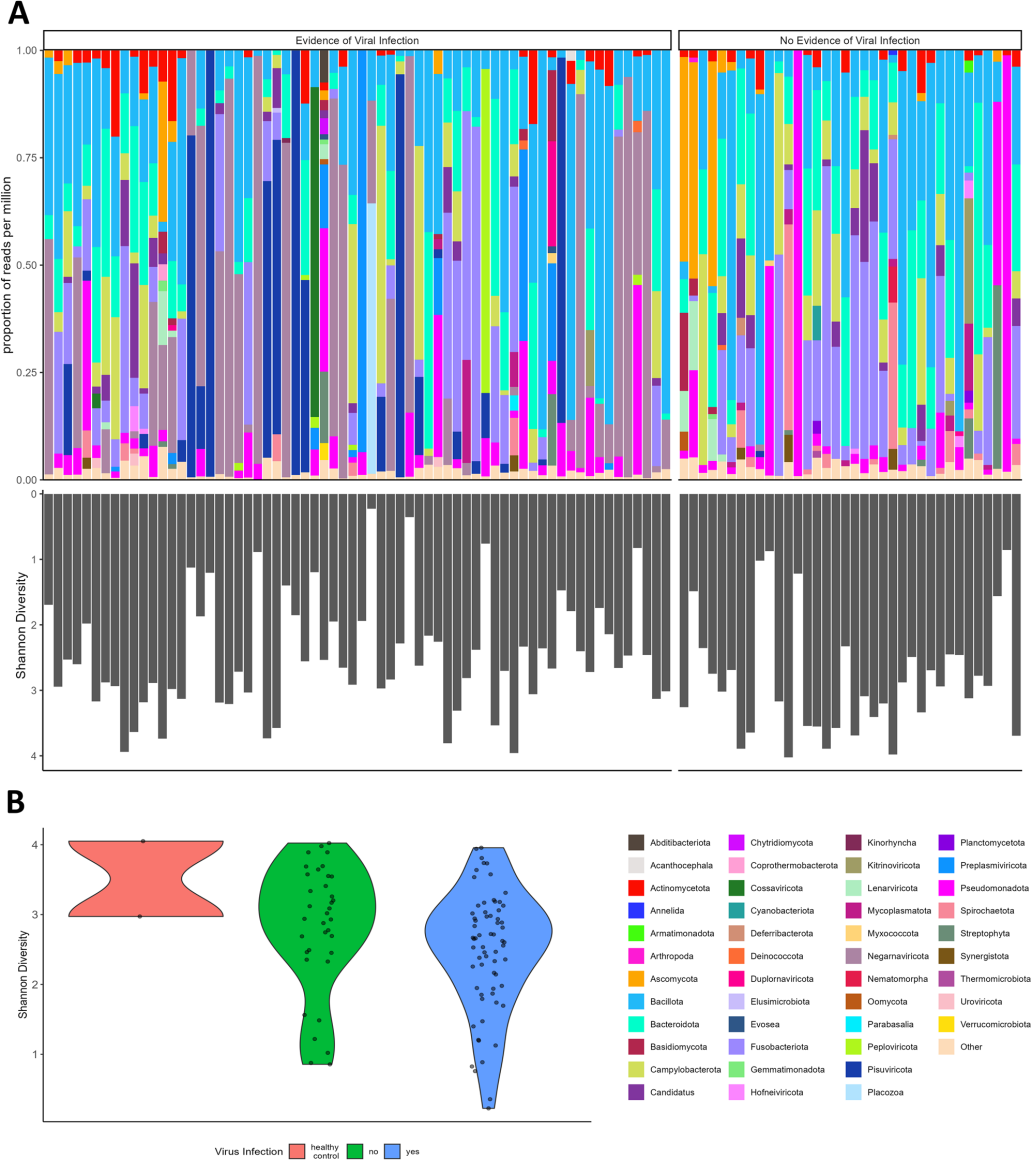
**A**) The relative proportion of reads per million contributed by each microbial class for each sample, stratified by whether or not evidence of viral infection was detected. Classes of microbes that contributed less than 1% of the relative proportion of total reads per million were reclassified as “Other.” The Shannon diversity index of each sample is displayed beneath each column. **B**) Shannon diversity distributions for healthy controls included in the analysis, and for samples in which evidence of viral infection was and was not found

A range of bacteria, eukaryotes, and viruses were identified in the nasopharyngeal swabs (Fig. 2). Although healthy controls (*n* = 2) had the highest average Shannon diversity index relative to both febrile samples with (*n* = 66) and without (*n* = 36) evidence of viral infection, and the mean Shannon diversity index was lowest among samples with evidence of viral infection, diversity differences between groups were not significant (ANOVA, *p* = 0.06).

### Viruses detected by mNGS

Evidence of infection with known pathogenic viruses was identified in 66 (66/102, 64.7%) nasopharyngeal samples, including from nine viral families (Table 2). When excluding the previously diagnosed positive controls, evidence of viral infection was identified in 57 samples (57/91, 62.6%). Evidence of multiple virus coinfection was found in 16 (16/66, 24%) of the samples with evidence of infection, including two positive controls. Most viral coinfections (*n* = 12) included two viruses, though three samples had coinfections with three viruses, and one sample had evidence of infection with five viruses (bocaparvovirus, mastadenovirus, betapolyomavirus, cytomegalovirus, and alphacoronavirus) (see Table S3 for further details).

**Table 2:** Viruses identified by mNGS in nasopharyngeal samples that meet the threshold for evidence of viral infection. Samples were collected in public hospitals across Madagascar between 2014 and 2019, screened negative on multiplex RT-qPCR, and underwent sequencing

Virus	Family	Number of Samples*	Number of Whole Genomes
Betapolyomavirus	*Polyomaviridae*	1	-
Bocaparvovirus	*Parvoviridae*	2	1
Coronavirus	*Coronaviridae*	6	2
Betacoronavirus		5	2
Alphacoronavirus		1	-
Cytomegalovirus	*Orthoherpesviridae*	8	-
Enterovirus	*Picornaviridae*	20	11
Rhinovirus A		5	5
Rhinovirus B		1	1
Rhinovirus C		5	5
Other enteroviruses		9	-
Influenza virus	*Orthomyxoviridae*	3	-
Alphainfluenza virus		2	
Betainfluenza virus		1	
Mastadenovirus	*Adenoviridae*	12	-
Metapneumovirus	*Pneumoviridae*	8	3
Orthopneumovirus	*Pneumoviridae*	24	12
Parainfluenza virus	*Paramyxoviridae*	2	1
Human Parainfluenza virus 4		1	1
Human Parainfluenza virus 1		1	-
Rotavirus	*Reoviridae*	1	-
Simplexvirus	*Orthoherpesviridae*	1	-

* Does not sum to *n* = 66 samples with evidence of viral infection because 16 individuals were co-infected

Eleven positive controls were included in this analysis, comprising two patients with influenza A(H3N2) virus detection by RT-qPCR, eight patients diagnosed with RSV, and one with RSV-HRV coinfection. Concordance between mNGS and RT-qPCR methods was high (9/11 samples, 81.8%). Among the two positive controls initially diagnosed with A(H3N2) virus, mNGS identified evidence of alphainfluenzavirus infection in only one. Among the 8 positive controls initially diagnosed with RSV, mNGS identified evidence of RSV infection in all cases. In the RSV-HRV coinfected positive control, mNGS identified evidence of RSV but not HRV infection.

**Fig. 3:**
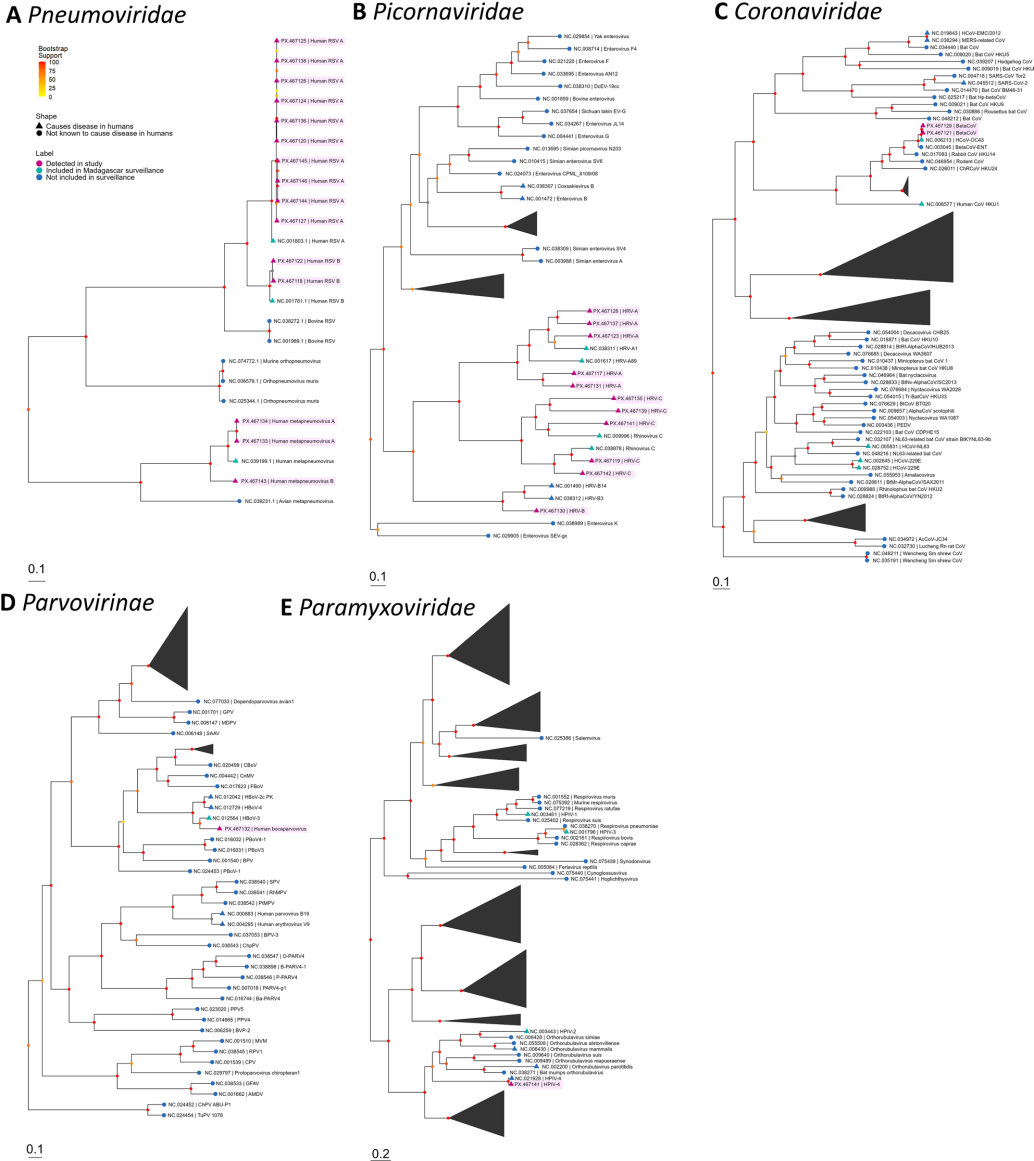
Phylogenetic characterization of the consensus genomes identified via mNGS including full genome nucleotide trees for **A**) *Pneumoviridae*, **B**) *Picornaviridae*, **C**) *Coronaviridae*, **D**) *Parvovirinae*, and **E**) *Paramyxoviridae*. Contextual sequences were obtained from NCBI’s RefSeq database, comprising reference sequences of the family of viruses identified in the Madagascar study. Maximum likelihood reconstruction of phylogenetic trees was conducted using GTR + I+G4 nucleotide substitution models. Tip color indicates whether viruses were detected by mNGS, included in the original multiplex-PCR screening protocol, or not included in the original surveillance. Text highlights also indicate viruses detected by mNGS. Tip shape indicates whether the virus is known to cause disease in humans. Node color indicates the bootstrap value obtained using Felsenstein’s method until convergence

Thirty consensus genomes were obtained from 29 of 66 samples with evidence of viral infection; consensus genomes corresponded to 12 (12/30, 40%) orthopneumoviruses (respiratory syncytial virus, RSV), 11 (11/30, 36.7%) rhinoviruses (HRV), 3 (3/30, 10%) metapneumoviruses (HMPV), 2 (2/30, 6.7%) betacoronaviruses, and 1 (1/30, 3.3%) each of bocavirus (HBoV) and parainfluenza virus (HPIV). Within each virus family, consensus genomes represented a broad virus diversity, including both RSV subtypes A and B; HRV species A, B, and C; human betacoronavirus species HKU1 and OC43; and HMPV subtypes 1 and 2 (Fig. 3a-e). All these viruses except HPIV-4 were included in the 14-pathogen surveillance panel, although primer match varied among the sequences (Figure S2; virus-specific mismatches described in the following section).

We describe the identified viruses in further detail:

#### Pneumoviridae

Multiple known human pathogens are members of the family *Pneumoviridae*, such as human metapneumovirus (HMPV) and human orthopneumovirus (RSV), both of which were included in the RT-qPCR surveillance panel. Between 2015 and 2017, 8 human metapneumovirus infections were identified by mNGS in our sample set from Madagascar, yielding three whole genome HMPV sequences, two HMPV strain A and one HMPV strain B. BLASTN comparison identified that HMPV-A sequences were closest to sequences circulating in China and Australia in the early 2010s and the HMPV-B sequence most resembled sequences circulating in Europe and east Asia in the same period. Although human metapneumovirus was included in the surveillance panel, both HMPV-A sequences had two nucleotide mismatches to the primer set (Figure S2).

**Fig. 4:**
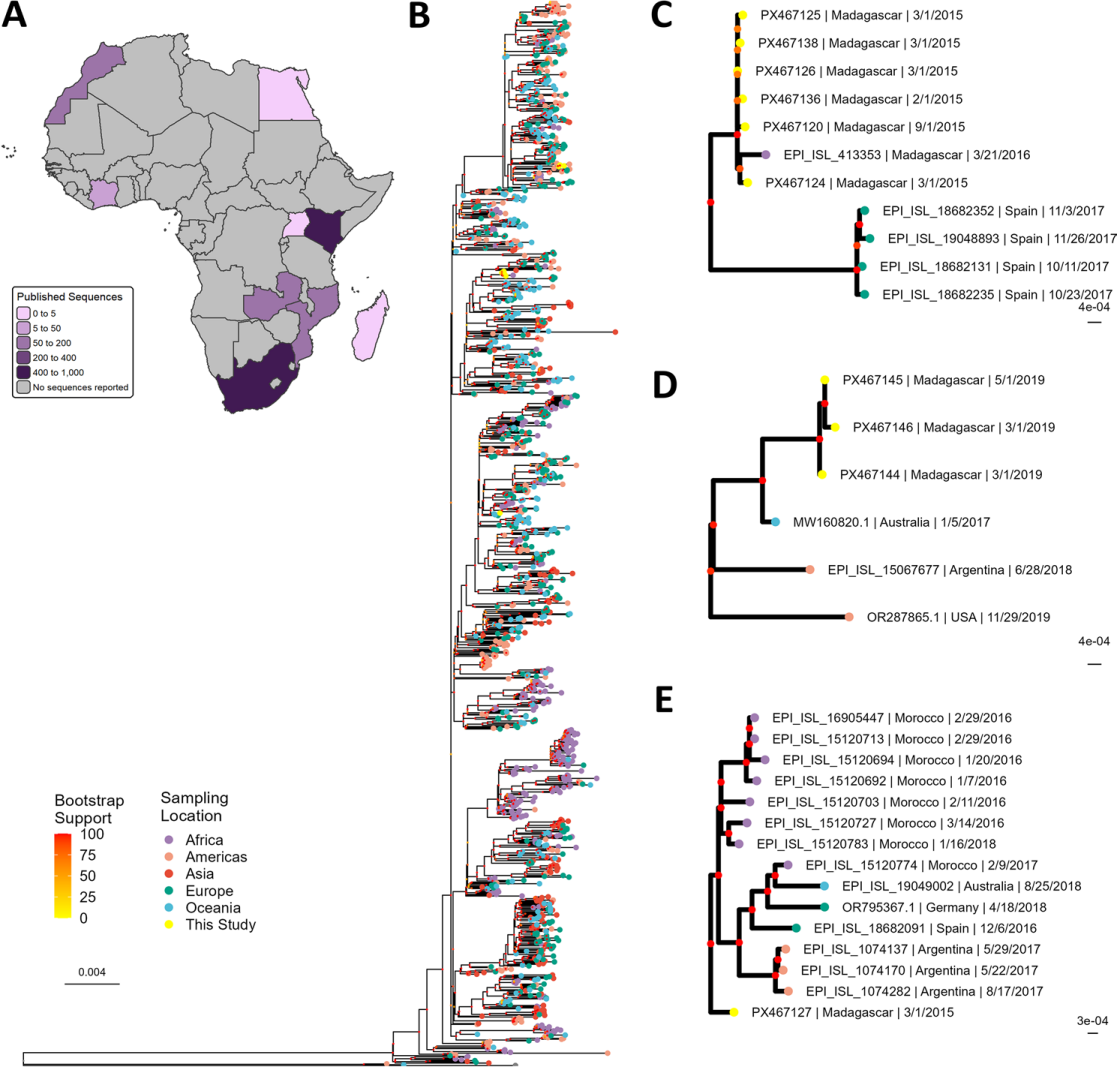
**A**) Map of all whole genome African RSV sequences available on GenBank/GISAID with collection date between 2013 and 2019. **B**) Maximum likelihood phylogeny (GTR + I+G4) of whole genome nucleotide RSV subtype A sequences (*n* = 1294) with tip color coded by continent of origin and node color indicating bootstrap value from 1,000 replicates. RSV-A sequences were obtained from public databases and subsampled. The reference sequence for RSV subtype B was used as an outgroup. **C**) Subset of part B phylogeny showing a cluster of Madagascar sequences from this study collected in 2015. **D**) Subset of the part B phylogeny including cluster of Madagascar sequences from this study collected in 2019. **E**) Subset of part B phylogeny including Madagascar sequence from this study collected in 2015

Twenty-four RSV infections were identified in the mNGS data, twelve of which yielded whole genome consensus sequences, including ten of subtype A and two of subtype B. No complete RSV sequence had perfect compatibility with the primer set used in RT-qPCR screening, with mismatches of at least one nucleotide per sequence. All of the subtype A sequences diverged from the forward primer one base from the 3’ end and all subtype B sequences differed from the 3’ end of the probe, the region most sensitive to misalignment in PCR surveillance [49] (Figure S2). Phylogenetic analysis of the RSV-A sequences indicated two distinct clusters of cases, collected in March 2015 (*n* = 5) and 2019 (*n* = 4) (Fig. 4a, b). The 2015 sequences clustered with a previously published sequence from Madagascar collected in early 2016, and, at a more basal node, with sequences collected in Europe (Fig. 4c). The 2019 sequences were related most closely to sequences circulating in Australia and the Americas between 2017 and 2019 (Fig. 4d). One 2015 Madagascar sequence identified in this study did not cluster with any other Madagascar sequences, but rather with sequences recovered from Morocco and Australia in subsequent years (2016, 2019) (Fig. 4e).

#### Picornaviridae

The family *Picornaviridae* includes enteroviruses, many of which are known to cause human infection with a range of symptoms. Human rhinovirus was the only enterovirus included in the multiplex PCR, and one positive control was initially diagnosed with an HRV-RSV coinfection. However, the HRV infection diagnosed by RT-qPCR was not detected by mNGS. Between 2014 and 2019, eleven rhinovirus infections were identified in the mNGS data, comprising five HRV-A sequences, five HRV-C sequences, and one HRV-B sequence. Primer matching was low for the HRV-B sequences and varied for HRV-A and HRV-C, although HRV-A and HRV-C more frequently had discrepancies at the 3’ end of the probe (Figure S2). HRV sequences do not appear to be closely related; among HRV-A, the Madagascar sequences were most closely related to strains A51, 21, 54, 94, and 101 while HRV-C sequences resembled types 3, 22, 24, and 27. Additionally, although no consensus genome was obtained, evidence of infection with enterovirus C99 and with coxsackie virus A was identified in one patient each (see Table S3).

#### Coronaviridae

The family *Coronaviridae* comprises many known human pathogens, including seasonal human coronaviruses as well as zoonotic coronaviruses with pandemic potential (e.g. SARS-CoV, MERS-CoV, SARS-CoV-2). Evidence of infection with both betacoronaviruses, including HCoV-OC43 and HCoV-HKU1, and the alphacoronavirus HCoV-NL63 was identified in six patients, yielding two consensus genomes of HCoV-OC43 (see Table S3). All human coronaviruses known to date were included in the surveillance panel. PCR primers perfectly aligned with one of the HCoV-OC43 full genomes identified (accession: PX467129) and mismatched by one nucleotide with the other (accession: PX467121) (Figure S2).

#### Parvovirinae

*Parvovirinae* includes known pathogens human bocaviruses 1, 2, 3, and 4. By mNGS, one human bocaparvovirus was identified in Madagascar from 2015. Although human bocavirus was included in the RT-qPCR screening, it was only detected by mNGS, and there were no mismatches between the primers and the sequence.

#### Paramyxoviridae

The viral family *Paramyxoviridae* also encompasses many known human pathogens, including measles virus, mumps, and human parainfluenza viruses. Of the four types of HPIV, only types HPIV-1, HPIV-2, and HPIV-3 were included in the multiplex RT-qPCR screening. Despite its inclusion in the surveillance panel, HPIV-1 was not detected in a PCR-negative sample from 2017, but was identified by mNGS, although a full genome sequence was not obtained. One full-genome human parainfluenza virus 4 was identified from a sample collected in 2016; this type of HPIV was not included in the surveillance panel. By BLASTN comparison, the HPIV-4 sequence was most similar to viruses from South America and Europe sampled between 2013 and 2016.

#### Polyomaviridae

Evidence of infection with human polyomavirus 4 (betapolyomavirus) was identified in one patient whose samples contained multiple co-detected viruses. This virus, first described in 2007 among hospitalized patients with pneumonia in Australia [50], has not previously been reported in Madagascar.

#### Orthoherpesviridae

*Orthoherpesviridae* is a family of DNA viruses capable of latent infection and subsequent reactivation, causing a wide range of diseases. No virus of this family was included in the multiplex RT-qPCR; however, two different viruses were detected by mNGS. One sample showed evidence of infection with herpes simplexvirus 1 (HSV-1) and eight samples with cytomegalovirus, although full genome coverage was not achieved for any of these viruses. The HSV-1 infection and all cases of cytomegalovirus were detected alongside at least one other virus.

#### Reoviridae

No *Reoviridae*, a viral family that includes many common causes of enteric and respiratory disease, were included in the surveillance screening. Using mNGS, a rotavirus was detected in one sample that also had evidence of infection with orthopneumovirus and mastadenovirus.

#### Adenoviridae

Human adenovirus was included in the multiplex RT-qPCR screening but not identified by PCR in any samples assayed. Nonetheless, using mNGS, we identified evidence of infection with mastadenovirus in twelve patients but were unable to obtain a full genome.

## Discussion

We identified several viral pathogens associated with respiratory febrile illness in Antananarivo, Madagascar that were not diagnosed at the time of initial hospital visit. All viruses for which we obtained consensus genomes were known human pathogens and most viruses identified in this study had been previously detected in Madagascar, including RSV [9, 51, 52], and influenza virus [6, 7, 52], both of which are responsible for high burdens of respiratory disease and hospitalization in children under five. Additionally, metapneumovirus [16, 53], bocaparvovirus [7, 53], seasonal coronaviruses [7], parainfluenza virus [7, 8], adenoviruses [54], rotavirus [54], and rhinovirus [6] and other enteroviruses, including enterovirus C99 [55] have all been identified in Madagascar during the last several decades. We present, to our knowledge, the first evidence of infection with human polyomavirus 4 in Madagascar. Despite prior documentation of their circulation in the country, neither cytomegalovirus, EV-C99 nor HPIV-4 were included in the 14-pathogen surveillance panel used for original screening. Rotavirus, which more frequently results in gastrointestinal disease manifestations but has been associated with respiratory symptoms [56], and herpes simplex virus 1, a virus which often establishes a latent infection not typically associated with respiratory symptoms but can be detected in instances of coinfection with another respiratory pathogen [57], a pattern replicated here, were additionally not included in the 14-pathogen original surveillance screening platform. Thus, while the majority of viruses identified by mNGS in our study commonly cause respiratory disease in humans and are likely of primary clinical relevance, some of these infections (e.g. cytomegalovirus, HSV-1) are less frequently associated with respiratory symptoms and more likely to occur coincidentally with another respiratory virus infection. As such, attributing disease to any specific pathogen remains difficult due to the possibility of latent infection, reactivation, or bystander detection of a virus in the sample from a febrile patient.

Our virus detection results are derived from a non-representative study population comprised only of hospitalized patients who went undiagnosed by the national surveillance program; nonetheless they largely aligned with previous research in the country. Consistent with global patterns of respiratory virus infections and previous estimates of the respiratory virus burden in Madagascar [7, 9], respiratory virus infections were detected at higher frequency among younger patients, a pattern which held even after excluding RSV infection, which is strongly associated with patient ages < 2 years in Madagascar [9]. We identified no pattern of viral infection by sex, also consistent with previous research in the country [7, 58]. Codetection of respiratory pathogens has also been previously reported at a similar rate in Malagasy patients [8]. Finally, the broad diversity of respiratory viruses identified in this study is in line with previous work from Madagascar that found a number of different pathogens which underpin care seeking for respiratory symptoms [8, 16, 59]. Studies in neighboring Reunion [60] island and other countries in sub-Saharan Africa likewise report detection of numerous types of viruses [61, 62] and wide intra-virus diversity [63] associated with respiratory illness in hospitals and clinics.

Despite a survey of microbial diversity in the remaining nasopharyngeal samples that did not yield evidence of viral infection (*n* = 36, 35.3%), we were unable to identify likely causal pathogens. Because many bacteria are not obligately pathogenic, without a more nuanced background sample of Malagasy populations to characterize the expected presence of nasopharyngeal microbial taxa, we cannot confidently conclude that the presence of a pathogen or dominance in the sequencing reads corresponds to a febrile illness. While previous work has characterized human respiratory tract microbiomes, microbial profiles of nasopharyngeal microbiomes can vary widely [64]. Further, the respiratory microbiome has been shown to change throughout an individual’s life [65] as well as in response to a number of behavioral or environmental factors [65, 66]. Previous studies have addressed the inherent variation in respiratory microbiomes by establishing thresholds for attributing respiratory illness to bacterial infection (i.e. in comparison to robust background selections of microbiome composition [17, 67]) or associated particular biotypes of healthy and infected respiratory microbiomes [68]. However, this study collected nasopharyngeal samples from only two healthy controls, and we were unable to establish statistically significant changes in bacterial presence in nasopharyngeal samples. By contrast, respiratory viruses identified in this study are largely not considered potential commensal organisms, and we conclude their presence in a nasopharyngeal sample corresponds to recent infection.

Using a global dataset of RSV subtype A sequences circulating concurrently with our Madagascar sequences, we identified evidence of both global interconnectivity and local transmission. The phylogenetic clustering of five RSV sequences collected in early 2015 with a previously published sequence from Madagascar suggests ongoing transmission within the country, in contrast with the divergent 2019 sequence cluster, which appears to have arrived to Madagascar via a different international transmission event. While thousands of human orthopneumovirus sequences have been made public from upwards of twenty African nations, complete or nearly complete RSV sequences of any subtype collected before 2020 are publicly reported in NCBI and GISAID databases from only nine countries. The COVID-19 pandemic resulted in a global expansion in sequencing technology [69], and since 2020, sequencing output of RSV has increased, with additional countries reporting thousands more sequences. However, limited sampling density of global RSV cases during our study interval restricts inference corresponding to the exact dynamics of RSV introductions to Madagascar. Expanded sequencing pipelines and public reporting of RSV will permit analysis of source and directionality of transmission.

Many of the viral pathogens identified by mNGS techniques were included in the multiplex RT-qPCR panel for screening respiratory infections at Malagasy hospitals. It is possible that a mismatch between the specific primers involved in the RT-qPCR and the genomic composition of circulating pathogens at the time of study resulted in the surveillance system missing detection of these infections; mismatches within a few bases of the 3’ end of a primer sequence are known to reduce assay sensitivity [49], and such mismatches have previously resulted in false negatives in screenings for RNA viruses including RSV [70] and influenza [71]. Further, the substantial within-taxa diversity of viral genomes could result in reduced sensitivity of PCR methods. Pairwise identity between primers and sequences varied among the sequences identified in this study, with most virus sequences diverging from the primer sequences. As a targeted method, RT-qPCR is more sensitive than mNGS, which can miss low viral load samples, an effect somewhat ameliorated by our decision to adopt a relatively relaxed threshold for determining evidence of viral infection, which identified infections in most previously diagnosed positive controls. It is possible, however, that our chosen thresholds could have detected older or incidental viral infections than those directly responsible for fever, thus limiting our ability to infer clinically relevant pathogens. Nonetheless, mNGS’s reduced sensitivity compared to RT-qPCR could explain the two positive control viruses previously detected by PCR that were not identified by mNGS. Despite these nuances, our findings largely underscore the importance of multiplex surveillance for respiratory pathogens and support ongoing surveillance efforts in Madagascar, as the majority of viruses identified by mNGS were represented in the 14-virus multiplex RT-qPCR screening panel. Four of the pathogens identified via mNGS that are not included in this panel, cytomegalovirus, EVC-99, HPIV-4 and rotavirus, have also been previously identified, albeit at low frequency, in Madagascar [8, 55].

Evidence of viral infection was identified in approximately two thirds of patient samples included in this study with many samples still lacking diagnosis. Previous studies using mNGS to identify causes of undiagnosed illness have reported a range of pathogen detection rates, with some studies mirroring our detection rates with pathogen identification reported in close to 60% of samples [17, 19], while others report pathogen identification in < 50% of samples [18, 21, 72], and others still demonstrate that higher detection rates are also possible [73]. While it is likely that some of the still-unidentified respiratory illness in our dataset can be attributed to bacterial or eukaryotic infections, it is also possible that causative viral infections went unrecognized by mNGS. Contrary to previous findings that viral infection reduces nasopharyngeal microbiome diversity [67, 74], we did not find a statistically significant difference in diversity indices between samples with and without evidence of viral infection, potentially suggesting that our thresholds for determining respiratory infection were too narrow, or that the mNGS did not capture all viral infections within the cohort. If samples were collected after the onset of infection and illness, false-negative results are possible as viruses may no longer be detectable either through PCR or sequencing at a later timepoint in an individual’s infection, particularly given that many of the viruses identified in this study are RNA viruses, which degrade rapidly. Previous qualitative work focused on malarial fever in Madagascar suggests that care-seeking is frequently delayed until either the sick person does not respond to home treatment or the illness becomes severe [75]. Such delays could reduce the probability of virus detection from febrile disease. In previous work using mNGS to study SARS-CoV-2, deep sequencing was not always able to identify genetic material in samples that had tested positive via PCR, with detection most successful during early stage disease [67]. Additionally, lack of maintenance of cold chain risks degradation of RNA in samples and correspondingly limited detection by molecular methods, a possibility that cannot be ruled out here. It is also possible that those reporting to a hospital with fever and respiratory symptoms could have an infection that is not primarily shed in respiratory excreta. Previous work suggests investigation of blood, serum, and fecal samples may yield more complete insight into currently undiagnosed febrile patients presenting in Madagascar hospitals; blood samples from individuals with fever enrolled between 2011 and 2013 in Madagascar analyzed with qPCR identified bacterial, eukaryotic, and viral pathogens, including *Klebsiella pneumoniae*, *Candida* spp., and dengue, enterovirus, and cytomegalovirus, but were only able to “diagnose” a pathogen in 25% of samples [76]. Future work leveraging metagenomic sequencing methods on a wider range of samples with associated metadata may begin to bridge this remaining gap in pathogen detection and provide deeper insight into fever of unknown cause in Madagascar.

## Conclusion

A variety of respiratory viruses contribute to some, but not all, undiagnosed healthcare visits for fever and cough in Madagascar’s capital, Antananarivo. While the 14-pathogen multiplex PCR test used for pathogen surveillance cannot detect additional circulating viruses and primer compatibility may have reduced the sensitivity of PCR tests to the viruses identified by mNGS, the surveillance platform for respiratory infections in Madagascar nonetheless appears well-suited to the current burden of disease. However, mNGS analysis of undiagnosed fevers provided a more comprehensive overview of the diversity of viruses resulting in hospital visits for febrile illness. Integrating mNGS with a shorter delay into disease surveillance efforts could provide a public health benefit in diagnosing pathogens not included in existing screening panels as well as a method for identifying novel or zoonotic pathogens emerging in human populations. Molecular epidemiology using mNGS provides a useful strategy to investigate the etiology of undiagnosed respiratory fevers and expanded sequencing capacity linked more closely with sample collection could yield further insight.

## Supplementary Information

Below is the link to the electronic supplementary material.

Supplementary Material 1

## Data Availability

The genomic datasets generated and analyzed during the current study are available in the Sequence Read Archive (BioProject number PRJNA1328692) and on GenBank (accession numbers PX467117-PX467146).

## Abbreviations

mNGS: Metagenomic Next Generation Sequencing
RT-qPCR: Reverse transcription quantitative polymerase chain reaction
RSV: Respiratory syncytial virus
RSV-A: Respiratory syncytial virus subtype A
IPM: Institut Pasteur de Madagascar
LMICs: Lower- and middle-income countries
HRV: Human rhinovirus
HMPV: Human metapneumovirus
HBoV: Human bocavirus
HPIV: Human parainfluenza virus
HSV: Herpes simplex virus
