# Supplementary material for: Identifying viral infections through metagenomic Next Generation Sequencing of undiagnosed respiratory fevers in Madagascar (2014–2019)
Source: BMC Infect Dis. 2026 Jun 6;26:1369. doi: 10.1186/s12879-026-13715-7 (PMC13386818; doi:10.1186/s12879-026-13715-7)

**Supplement**

**Text S1. Further details on mNGS pipeline analysis:**

To calculate the z-score for each taxon detected in a sample, the following formula is used

$$z= \frac{x-\mu}{\sigma}=\frac{\left( rpm of taxa T in sample S \right)-mean(rpm of taxa T in background model samples)}{std\_dev(rpm of taxa in background model samples)}$$

If a taxon was not present in the samples used to generate the background model, a z-score of 100 was used. Alternately, if a taxon is in the background model but not present in a sample, a z-score of -100 was used.

We used a background model generated from the two healthy controls included in this analysis and implemented in CZID.

**Table S1.** Pathogens included in RT-qPCR screening for febrile patients with respiratory symptoms at hospitals in Madagascar, additional details have been previously reported^7^.

| **Pathogens in Multiplex RT-qPCR:** | **NCBI Accession** |
| --- | --- |
| Human parainfluenza virus 1 (HPIV-1) | U70948 |
| Human parainfluenza virus 2 (HPIV-2) | D00865 |
| Human parainfluenza virus 3 (HPIV-3) | EU814626 |
| Human coronavirus OC43 (HCoV-OC43) | AY391777 |
| Human rhinovirus (HRV) | EU840952 |
| Betainfluenza virus | FJ152012 |
| Human metapneumovirus (HMPV) | AY145286 |
| Respiratory syncytial virus (RSV) | AY353550 |
| Alphainfluenza virus | DQ508860 |
| Human coronavirus 229E (HCoV-229E) | AF304460 |
| Human coronavirus HKU1 (HCoV-HKU1) | AY597011 |
| Human coronavirus NL63 (HCoV-NL63) | AY563107 |
| Human adenovirus (HAdV) | J01917 |
| Human bocavirus (HBoV) | DQ000495 |

**Table S2**. NCBI and GISAID accession numbers for each reference sequence included in the maximum likelihood phylogenies presented in **Figure 2** and **Figure 4**.

| Phylogeny | NCBI Accession Numbers |
| --- | --- |
| *Picornaviridae* (Figure 2. A) | NC_038306.1, NC_038307.1, NC_038308.1, NC_038309.1, NC_038310.1, NC_038311.1, NC_038312.1, NC_038878.1, NC_038989.1, NC_037654.1, NC_034267.1, NC_033695.1, NC_030454.1, NC_029905.1, NC_029854.1, NC_024073.1, NC_021220.1, NC_013695.1, NC_010415.1, NC_009996.1, NC_008714.1, NC_004441.1, NC_003988.1, NC_001612.1, NC_001617.1, NC_001472.1, NC_001490.1, NC_001430.1, NC_001859.1, NC_002058.3, NC_003987.1 |
| *Paramyxoviridae* (Figure 2. B) | NC_074766.1, NC_075392.1, NC_075404.1, NC_075438.1, NC_075439.1, NC_075440.1, NC_075441.1, NC_075446.1, NC_075983.1, NC_076003.1, NC_076269.1, NC_076397.1, NC_076558.1, NC_076570.1, NC_076597.1, NC_076605.1, NC_076624.1, NC_076625.1, NC_076941.1, NC_076951.1,  NC_077219.1, NC_055167.1, NC_055168.1, NC_055508.1, NC_043539.1, NC_043540.1, NC_040796.1, NC_038270.1, NC_038271.1, NC_039015.1, NC_039016.1, NC_039017.1, NC_039018.1, NC_039019.1, NC_039194.1, NC_039195.1, NC_039196.1, NC_039197.1, NC_039198.1, NC_039223.1, NC_039230.1, NC_034968.1, NC_030231.1, NC_028362.1, NC_028249.1, NC_025386.1, NC_025390.1, NC_025402.1, NC_025403.1, NC_025404.1, NC_025407.1, NC_025410.1, NC_025343.1, NC_025347.1, NC_025348.1, NC_025349.3, NC_025350.1, NC_025351.1, NC_025352.1, NC_025355.1, NC_025360.1, NC_025361.1, NC_025363.1, NC_025373.1, NC_025256.1, NC_021928.1, NC_019531.1, NC_017937.1, NC_009640.1, NC_009489.1, NC_007803.1, NC_007454.1, NC_006430.1, NC_006428.1, NC_006383.2, NC_006296.2, NC_005339.1, NC_005283.1, NC_005084.2, NC_005036.1, NC_003461.1, NC_004074.1, NC_002728.1, NC_002199.1, NC_002200.1, NC_002161.1, NC_001498.1, NC_001796.2, NC_001921.1, NC_001906.3, NC_001552.1, NC_003443.1, NC_025345.1 |
| *Pneumovirinae* (Figure 2. C) | NC_074772.1, NC_039199.1, NC_038272.1, NC_039231.1, NC_038235.1, NC_025344.1, NC_006579.1, NC_001989.1, NC_001781.1, NC_001803.1, NC_025345.1 |
| *Coronaviridae*  (Figure 2. D) | NC_054003.1, NC_054004.1, NC_054015.1, NC_055953.1, NC_076629.1, NC_076684.1, NC_076685.1, NC_076697.1, NC_048211.1, NC_048212.1, NC_048213.1, NC_048214.1, NC_048216.1, NC_048217.1, NC_046954.1, NC_046955.1, NC_046964.1, NC_046965.1, NC_045512.2, NC_006213.1, NC_011547.1, NC_038294.1, NC_038861.1, NC_039207.1, NC_039208.1, NC_035191.1, NC_034972.1, NC_034440.1, NC_032730.1, NC_032107.1, NC_030886.1, NC_030292.1, NC_028752.1, NC_028806.1, NC_028811.1, NC_028814.1, NC_028824.1, NC_028833.1, NC_026011.1, NC_025217.1, NC_023760.1, AC_000192.1, NC_022103.1, NC_019843.3, NC_018871.1, NC_017083.1, NC_016991.1, NC_016992.1, NC_016993.1, NC_016994.1, NC_016995.1, NC_016996.1, NC_014470.1, NC_012936.1, NC_011549.1, NC_011550.1, NC_010800.1, NC_010646.1, NC_010437.1, NC_010438.1, NC_009988.1, NC_009657.1, NC_009019.1, NC_009020.1, NC_009021.1, NC_006577.2, NC_005831.2, NC_004718.3, NC_003045.1, NC_003436.1, NC_002645.1, NC_002306.3, NC_001846.1, NC_001451.1, NC_022787.1 |
| *Parvovirinae*  (Figure 2. E) | NC_077033.1, NC_038533.1, NC_038535.1, NC_038536.1, NC_038539.1, NC_038540.1, NC_038541.1, NC_038542.1, NC_038543.1, NC_038545.1, NC_038546.1, NC_038547.1, NC_038898.1, NC_037053.1, NC_029797.1, NC_024452.1, NC_024453.2, NC_024454.1, NC_023020.1, NC_020499.1, NC_017823.1, NC_016744.1, NC_016031.1, NC_016032.1, NC_014665.1, NC_014468.1, NC_012729.2, NC_012564.1, NC_012042.1, NC_007018.1, NC_006259.1, NC_006260.1, NC_006261.1, NC_006152.1, NC_006147.2, NC_006148.1, NC_005889.1, NC_004442.1, NC_004295.1, NC_000883.2,  NC_002077.1, NC_001829.1, NC_001729.1, NC_001701.1, NC_001539.1, NC_001540.1, NC_001510.1, NC_001662.1 |
| RSV subtype A GISAID Accession Numbers  (Figure 4. B) | EPI_ISL_1074035, EPI_ISL_1074035, EPI_ISL_2582644, EPI_ISL_19049203, EPI_ISL_19048415, EPI_ISL_1074036, EPI_ISL_1074036, EPI_ISL_2578813, EPI_ISL_19048345, EPI_ISL_19048214, EPI_ISL_1074270, EPI_ISL_1074270, EPI_ISL_2595607, EPI_ISL_19048918, EPI_ISL_19048920, EPI_ISL_1074267, EPI_ISL_1074267, EPI_ISL_2595477, EPI_ISL_19049181, EPI_ISL_19048798, EPI_ISL_1074190, EPI_ISL_1074190, EPI_ISL_2595711, EPI_ISL_18090692, EPI_ISL_19048886, EPI_ISL_1074124, EPI_ISL_1074124, EPI_ISL_2578825, EPI_ISL_1834083, EPI_ISL_19048467, EPI_ISL_1074039, EPI_ISL_1074039, EPI_ISL_2582239, EPI_ISL_1653950, EPI_ISL_19048056, EPI_ISL_1074187, EPI_ISL_1074187, EPI_ISL_2578826, EPI_ISL_1834084, EPI_ISL_19047995, EPI_ISL_1074108, EPI_ISL_1074108, EPI_ISL_2582253, EPI_ISL_1834085, EPI_ISL_19048985, EPI_ISL_1074105, EPI_ISL_1074105, EPI_ISL_2582841, EPI_ISL_1653954, EPI_ISL_19048386, EPI_ISL_1074271, EPI_ISL_1074271, EPI_ISL_2595681, EPI_ISL_1653955, EPI_ISL_19048786, EPI_ISL_1074040, EPI_ISL_1074040, EPI_ISL_2578789, EPI_ISL_1653959, EPI_ISL_19048375, EPI_ISL_1074243, EPI_ISL_1074243, EPI_ISL_2582641, EPI_ISL_1834086, EPI_ISL_19048948, EPI_ISL_1074244, EPI_ISL_1074244, EPI_ISL_2595605, EPI_ISL_1834087, EPI_ISL_19049046, EPI_ISL_1074262, EPI_ISL_1074262, EPI_ISL_2582264, EPI_ISL_1834088, EPI_ISL_19048695, EPI_ISL_1074042, EPI_ISL_1074042, EPI_ISL_2582250, EPI_ISL_2156812, EPI_ISL_2543931, EPI_ISL_1074272, EPI_ISL_1074272, EPI_ISL_2595684, EPI_ISL_1834089, EPI_ISL_18482775, EPI_ISL_1074135, EPI_ISL_1074135, EPI_ISL_2578679, EPI_ISL_1653970, EPI_ISL_18482771, EPI_ISL_1074114, EPI_ISL_1074114, EPI_ISL_2582227, EPI_ISL_1653972, EPI_ISL_18482736, EPI_ISL_1074250, EPI_ISL_1074250, EPI_ISL_2582646, EPI_ISL_1834090, EPI_ISL_18482814, EPI_ISL_1074048, EPI_ISL_1074048, EPI_ISL_2595609, EPI_ISL_2156813, EPI_ISL_18482802, EPI_ISL_1074049, EPI_ISL_1074049, EPI_ISL_2582510, EPI_ISL_1834091, EPI_ISL_18482804, EPI_ISL_1074274, EPI_ISL_1074274, EPI_ISL_19049087, EPI_ISL_1653985, EPI_ISL_17964327, EPI_ISL_1074191, EPI_ISL_1074191, EPI_ISL_19049002, EPI_ISL_1653986, EPI_ISL_15753442, EPI_ISL_1074168, EPI_ISL_1074168, EPI_ISL_19049090, EPI_ISL_1653987, EPI_ISL_18482728, EPI_ISL_412857, EPI_ISL_15067677, EPI_ISL_1834133, EPI_ISL_1653988, EPI_ISL_18482801, EPI_ISL_1074170, EPI_ISL_15067678, EPI_ISL_1834138, EPI_ISL_1653990, EPI_ISL_18482746, EPI_ISL_1074171, EPI_ISL_15067679, EPI_ISL_1834140, EPI_ISL_1653992, EPI_ISL_18482766, EPI_ISL_1074137, EPI_ISL_15055316, EPI_ISL_1834141, EPI_ISL_1834093, EPI_ISL_18482805, EPI_ISL_1074060, EPI_ISL_15055317, EPI_ISL_1834142, EPI_ISL_1834094, EPI_ISL_18482815, EPI_ISL_1074160, EPI_ISL_15055318, EPI_ISL_1834144, EPI_ISL_1834096, EPI_ISL_2543816, EPI_ISL_1074281, EPI_ISL_15055319, EPI_ISL_1834145, EPI_ISL_1834097, EPI_ISL_6174135, EPI_ISL_412857, EPI_ISL_15055320, EPI_ISL_1834146, EPI_ISL_1834100, EPI_ISL_2543824, EPI_ISL_1074170, EPI_ISL_15055321, EPI_ISL_1834147, EPI_ISL_1834102, EPI_ISL_2543820, EPI_ISL_1074171, EPI_ISL_15055322, EPI_ISL_1834148, EPI_ISL_1834103, EPI_ISL_2543814, EPI_ISL_1074137, EPI_ISL_2595715, EPI_ISL_1834149, EPI_ISL_1834105, EPI_ISL_6174136, EPI_ISL_1074060, EPI_ISL_2582223, EPI_ISL_10914989, EPI_ISL_1834106, EPI_ISL_2543817, EPI_ISL_1074160, EPI_ISL_2582771, EPI_ISL_15895124, EPI_ISL_1834107, EPI_ISL_5522630, EPI_ISL_1074281, EPI_ISL_2582512, EPI_ISL_15895125, EPI_ISL_2156814, EPI_ISL_6495010, EPI_ISL_1074282, EPI_ISL_2578812, EPI_ISL_19049086, EPI_ISL_1834110, EPI_ISL_6494785, EPI_ISL_15067675, EPI_ISL_2543812, EPI_ISL_19048146, EPI_ISL_1834112, EPI_ISL_6494793, EPI_ISL_15067676, EPI_ISL_2543823, EPI_ISL_19048585, EPI_ISL_1834114, EPI_ISL_6494794, EPI_ISL_6494992, EPI_ISL_2543813, EPI_ISL_19048813, EPI_ISL_1834119, EPI_ISL_6494796, EPI_ISL_6494993, EPI_ISL_2543819, EPI_ISL_19048380, EPI_ISL_1834120, EPI_ISL_6494800, EPI_ISL_6494996, EPI_ISL_6208719, EPI_ISL_19048904, EPI_ISL_1834123, EPI_ISL_6494805, EPI_ISL_6494998, EPI_ISL_6494983, EPI_ISL_2543818, EPI_ISL_1834125, EPI_ISL_6494806, EPI_ISL_6495001, EPI_ISL_6494984, EPI_ISL_2543825, EPI_ISL_1834126, EPI_ISL_6494807, EPI_ISL_6495003, EPI_ISL_6494985, EPI_ISL_2543811, EPI_ISL_1834129, EPI_ISL_6494808, EPI_ISL_6495004, EPI_ISL_6494988, EPI_ISL_2543815, EPI_ISL_1834130, EPI_ISL_6494809, EPI_ISL_6495007, EPI_ISL_6494991, EPI_ISL_2543826, EPI_ISL_1834132, EPI_ISL_11428309, EPI_ISL_6495009, EPI_ISL_19048791, EPI_ISL_6494815, EPI_ISL_732369, EPI_ISL_6494811, EPI_ISL_19048764, EPI_ISL_19049084, EPI_ISL_6494816, EPI_ISL_732372, EPI_ISL_19048366, EPI_ISL_17995673, EPI_ISL_18789104, EPI_ISL_6494820, EPI_ISL_1520381, EPI_ISL_19049059, EPI_ISL_17995647, EPI_ISL_18789106, EPI_ISL_6494821, EPI_ISL_1647383, EPI_ISL_18788990, EPI_ISL_17995649, EPI_ISL_18789111, EPI_ISL_6494822, EPI_ISL_1647385, EPI_ISL_18788992, EPI_ISL_17995675, EPI_ISL_19048895, EPI_ISL_6494823, EPI_ISL_1647387, EPI_ISL_18788999, EPI_ISL_17995641, EPI_ISL_19048903, EPI_ISL_10954007, PI_ISL_11428318, EPI_ISL_18789028, EPI_ISL_17995764, EPI_ISL_19048430, EPI_ISL_732337, EPI_ISL_1520390, EPI_ISL_19063408, EPI_ISL_17995651, EPI_ISL_19048134, EPI_ISL_732338, EPI_ISL_412865, EPI_ISL_19063407, EPI_ISL_19048900, EPI_ISL_19048107, EPI_ISL_732340, EPI_ISL_412866, EPI_ISL_19063403, EPI_ISL_19048393, EPI_ISL_17995640, EPI_ISL_732342, EPI_ISL_1520414, EPI_ISL_19063401, EPI_ISL_19063415, EPI_ISL_17995657, EPI_ISL_732344, EPI_ISL_1520419, EPI_ISL_19049182, EPI_ISL_19063400, EPI_ISL_17995663, EPI_ISL_732345, EPI_ISL_1520423, EPI_ISL_19048789, EPI_ISL_19063394, EPI_ISL_17995652, EPI_ISL_732359, EPI_ISL_1647400, EPI_ISL_19048902, EPI_ISL_19063391, EPI_ISL_17995642, EPI_ISL_732360, EPI_ISL_1520436, EPI_ISL_19048044, EPI_ISL_19063390, EPI_ISL_17995644, EPI_ISL_732361, EPI_ISL_19048255, EPI_ISL_2578731, EPI_ISL_19063387, EPI_ISL_17995643, EPI_ISL_732368, EPI_ISL_19048782, EPI_ISL_2578732, EPI_ISL_19063381, EPI_ISL_17995645, EPI_ISL_19048535, EPI_ISL_2578776, EPI_ISL_2578733, EPI_ISL_19063380, EPI_ISL_17995646, EPI_ISL_19047968, EPI_ISL_2582866, EPI_ISL_2582157, EPI_ISL_19063378, EPI_ISL_17995674, EPI_ISL_19049099, EPI_ISL_2595540, EPI_ISL_2582160, EPI_ISL_19063377, EPI_ISL_2578807, EPI_ISL_19048066, EPI_ISL_2595541, EPI_ISL_18682315, EPI_ISL_19063369, EPI_ISL_2582567, EPI_ISL_19048638, EPI_ISL_2578806, EPI_ISL_18682128, EPI_ISL_19063411, EPI_ISL_2578818, EPI_ISL_19048783, EPI_ISL_2595548, EPI_ISL_18682345, EPI_ISL_19063364, EPI_ISL_2582673, EPI_ISL_2543828, EPI_ISL_2595550, EPI_ISL_18682317, EPI_ISL_19063360, EPI_ISL_2595636, EPI_ISL_2543830, EPI_ISL_2582565, EPI_ISL_18682085, EPI_ISL_19063358, EPI_ISL_2582260, EPI_ISL_2543831, EPI_ISL_1647457, EPI_ISL_18682346, EPI_ISL_19063409, EPI_ISL_2578808, EPI_ISL_2543835, EPI_ISL_1647458, EPI_ISL_18682089, EPI_ISL_413222, EPI_ISL_2578821, EPI_ISL_2543838, EPI_ISL_1647459, EPI_ISL_18682436, EPI_ISL_2543761, EPI_ISL_2582681, EPI_ISL_2543839, EPI_ISL_1647460, EPI_ISL_18682437, EPI_ISL_2543827, EPI_ISL_2582147, EPI_ISL_2543840, EPI_ISL_1647461, EPI_ISL_18682366, EPI_ISL_2543841, EPI_ISL_15120774, EPI_ISL_15120782, EPI_ISL_1647462, EPI_ISL_18682268, EPI_ISL_2543842, EPI_ISL_15120780, EPI_ISL_15120778, EPI_ISL_1647463, EPI_ISL_18682404, EPI_ISL_2543843, EPI_ISL_15120781, EPI_ISL_15120688, EPI_ISL_1647464, EPI_ISL_18682088, EPI_ISL_2543844, EPI_ISL_15120775, EPI_ISL_15120785, EPI_ISL_1647465, EPI_ISL_18682417, EPI_ISL_15120776, EPI_ISL_1647456, EPI_ISL_16905448, EPI_ISL_1647466, EPI_ISL_18682269, EPI_ISL_15120777, EPI_ISL_15120772, PI_ISL_15120786, EPI_ISL_1647467, EPI_ISL_18682443, EPI_ISL_15120678, EPI_ISL_15120675, EPI_ISL_15120679, EPI_ISL_1647468, EPI_ISL_18682431, EPI_ISL_15753534, EPI_ISL_15120671, EPI_ISL_15120692, EPI_ISL_1647469, EPI_ISL_18682367, EPI_ISL_15753488, EPI_ISL_15120672, EPI_ISL_15120690, EPI_ISL_17308687, EPI_ISL_18682090, EPI_ISL_15753565, EPI_ISL_15120771, EPI_ISL_15120681, EPI_ISL_17308688, EPI_ISL_18682271, EPI_ISL_15753435, EPI_ISL_16905447, EPI_ISL_15120689, EPI_ISL_17308690, EPI_ISL_18682130, EPI_ISL_15753558, EPI_ISL_15120713, EPI_ISL_15120682, EPI_ISL_17308691, EPI_ISL_18682439, EPI_ISL_15753474, EPI_ISL_15120783, EPI_ISL_15120683, EPI_ISL_17308693, EPI_ISL_18682420, EPI_ISL_15753375, EPI_ISL_15120719, EPI_ISL_15120677, EPI_ISL_17308694, EPI_ISL_18682272, EPI_ISL_15753271, EPI_ISL_15120716, EPI_ISL_15120721, EPI_ISL_17308696, EPI_ISL_18682347, EPI_ISL_19048936, EPI_ISL_15120729, EPI_ISL_15120694, EPI_ISL_17308699, EPI_ISL_18682229, EPI_ISL_19047972, EPI_ISL_15120727, EPI_ISL_15120723, EPI_ISL_17308701, EPI_ISL_18682407, EPI_ISL_19048878, EPI_ISL_15120717, EPI_ISL_15120665, EPI_ISL_17308703, EPI_ISL_18682320, EPI_ISL_19048034, EPI_ISL_15120676, EPI_ISL_15120730, EPI_ISL_17308706, EPI_ISL_18682091, EPI_ISL_19048703, EPI_ISL_19206758, EPI_ISL_15120666, EPI_ISL_17308709, EPI_ISL_18682350, EPI_ISL_19049058, EPI_ISL_19206383, EPI_ISL_15120667, EPI_ISL_17308712, EPI_ISL_18682234, EPI_ISL_19206546, EPI_ISL_19207202, EPI_ISL_15120726, EPI_ISL_17308713, EPI_ISL_18682370, EPI_ISL_19206547, EPI_ISL_19206389, EPI_ISL_15120668, EPI_ISL_17308715, EPI_ISL_18682432, EPI_ISL_19206548, EPI_ISL_19206394, EPI_ISL_15120703, EPI_ISL_17308717, EPI_ISL_18682281, EPI_ISL_19206582, EPI_ISL_19206399, EPI_ISL_15120706, EPI_ISL_17308718, EPI_ISL_18682323, EPI_ISL_19206553, EPI_ISL_19206176, EPI_ISL_15120669, EPI_ISL_17308719, EPI_ISL_18682238, EPI_ISL_19206556, EPI_ISL_19206178, EPI_ISL_15120670, EPI_ISL_17308720, EPI_ISL_18682282, EPI_ISL_19206160, EPI_ISL_19206181, EPI_ISL_15120728, EPI_ISL_17308721, EPI_ISL_18682293, EPI_ISL_19206557, EPI_ISL_19206409, EPI_ISL_15120743, EPI_ISL_17308722, EPI_ISL_18682283, EPI_ISL_19206349, EPI_ISL_19206412, EPI_ISL_15120745, EPI_ISL_17308723, EPI_ISL_18682284, EPI_ISL_19206587, EPI_ISL_19206189, EPI_ISL_19206326, EPI_ISL_17308724, EPI_ISL_18682288, EPI_ISL_19206561, EPI_ISL_19206190, EPI_ISL_19206328, EPI_ISL_17308725, EPI_ISL_18682326, EPI_ISL_19206589, EPI_ISL_19206418, EPI_ISL_19206331, EPI_ISL_17308726, EPI_ISL_18682329, EPI_ISL_19206591, EPI_ISL_19206192, EPI_ISL_19206332, EPI_ISL_17308727, EPI_ISL_18682132, EPI_ISL_19206594, EPI_ISL_19206193, EPI_ISL_19206333, EPI_ISL_15753481, EPI_ISL_19423088, EPI_ISL_19206596, EPI_ISL_19206194, EPI_ISL_19206334, EPI_ISL_15753273, EPI_ISL_19423101, EPI_ISL_19207190, EPI_ISL_19206196, EPI_ISL_19207210, EPI_ISL_15753478, EPI_ISL_19423102, EPI_ISL_19207191, EPI_ISL_19206200, EPI_ISL_19206581, EPI_ISL_15753431, EPI_ISL_19423103, EPI_ISL_19206604, EPI_ISL_19206443, EPI_ISL_2544038, EPI_ISL_15753455, EPI_ISL_19423104, EPI_ISL_19206372, EPI_ISL_19206201, EPI_ISL_2544039, EPI_ISL_17417597, EPI_ISL_19423105, EPI_ISL_19206376, EPI_ISL_19207208, EPI_ISL_2544041, EPI_ISL_17417596, EPI_ISL_19423106, EPI_ISL_19207199, EPI_ISL_19206214, EPI_ISL_412458, EPI_ISL_17417578, EPI_ISL_19423107, EPI_ISL_19206564, EPI_ISL_19206485, EPI_ISL_2595691, EPI_ISL_17417579, EPI_ISL_12970423, EPI_ISL_19206567, EPI_ISL_19206486, EPI_ISL_2578777, EPI_ISL_17417599, EPI_ISL_17417591, EPI_ISL_19206757, EPI_ISL_19207209, EPI_ISL_2582271, EPI_ISL_17417589, EPI_ISL_19048438, EPI_ISL_19206575, EPI_ISL_19206219, EPI_ISL_2595717, EPI_ISL_17417581, EPI_ISL_19048081, EPI_ISL_19206886, EPI_ISL_19206221, EPI_ISL_2578778, EPI_ISL_412859, EPI_ISL_19048118, EPI_ISL_19206297, EPI_ISL_19206492, EPI_ISL_2578816, EPI_ISL_2582274, EPI_ISL_19048864, EPI_ISL_19206317, EPI_ISL_19206237, EPI_ISL_19137648, EPI_ISL_19048213, EPI_ISL_19049065, EPI_ISL_19206321, EPI_ISL_19206520, EPI_ISL_19423108, EPI_ISL_19442707, EPI_ISL_19048111, EPI_ISL_19206323, EPI_ISL_19206521, EPI_ISL_19423109, EPI_ISL_19442470, EPI_ISL_19090977, EPI_ISL_19048038, EPI_ISL_19206528, EPI_ISL_19423110, EPI_ISL_19442668, EPI_ISL_19090969, EPI_ISL_19049057, EPI_ISL_19206843, EPI_ISL_19423114, EPI_ISL_19442530, EPI_ISL_19090970, EPI_ISL_19048882, EPI_ISL_19206532, EPI_ISL_19423115, EPI_ISL_9003918, EPI_ISL_19090971, EPI_ISL_19049083, EPI_ISL_19206846, EPI_ISL_19423116, EPI_ISL_19048075, EPI_ISL_19090972, EPI_ISL_19049146, EPI_ISL_19206847, EPI_ISL_19423117, EPI_ISL_19048454, EPI_ISL_19090973, EPI_ISL_19048314, EPI_ISL_19206850, EPI_ISL_19423118, EPI_ISL_19049279, EPI_ISL_19090974, EPI_ISL_19049116, EPI_ISL_19206851, EPI_ISL_19423081, EPI_ISL_19048196, EPI_ISL_19090975, EPI_ISL_19048911, EPI_ISL_19206854, EPI_ISL_19423083, EPI_ISL_19049134, EPI_ISL_19090976, EPI_ISL_19049105, EPI_ISL_19206534, EPI_ISL_19423084, EPI_ISL_19047977, EPI_ISL_19048108, EPI_ISL_19048101, EPI_ISL_19206535, EPI_ISL_19423087, EPI_ISL_19049210, EPI_ISL_19048971, EPI_ISL_19048348, EPI_ISL_19206538, EPI_ISL_19423090, EPI_ISL_19048731, EPI_ISL_18682255, EPI_ISL_19048735, EPI_ISL_19206274, EPI_ISL_19423092, EPI_ISL_19048198, EPI_ISL_18682096, EPI_ISL_19049124, EPI_ISL_19206277, EPI_ISL_19423082, EPI_ISL_19049074, EPI_ISL_18682083, EPI_ISL_19049139, EPI_ISL_19206881, EPI_ISL_18682263, EPI_ISL_18682343, EPI_ISL_18682316, EPI_ISL_19048938, EPI_ISL_18682257, EPI_ISL_18682264, EPI_ISL_18682344, EPI_ISL_18682225, EPI_ISL_19048123, EPI_ISL_18682379, EPI_ISL_18682219, EPI_ISL_18682356, EPI_ISL_18682221, EPI_ISL_18682244, EPI_ISL_18682262, EPI_ISL_18682365, EPI_ISL_2595604, EPI_ISL_17964343, EPI_ISL_18682133, EPI_ISL_18143557, EPI_ISL_15753556, EPI_ISL_2543978, EPI_ISL_17964352, EPI_ISL_18682134, EPI_ISL_18143644, EPI_ISL_15753564, EPI_ISL_2587417, EPI_ISL_17964346, EPI_ISL_18682170, EPI_ISL_19125809, EPI_ISL_15753379, EPI_ISL_2587355, EPI_ISL_17964337, EPI_ISL_18682332, EPI_ISL_19125740, EPI_ISL_15753551, EPI_ISL_2587431, EPI_ISL_17964329, EPI_ISL_18682094, EPI_ISL_19125764, EPI_ISL_15753453, EPI_ISL_2587452, EPI_ISL_17964311, EPI_ISL_18682295, EPI_ISL_19125785, EPI_ISL_15753275, EPI_ISL_2543975, EPI_ISL_17964367, EPI_ISL_18682296, EPI_ISL_19125851, EPI_ISL_15753444, EPI_ISL_2587494, EPI_ISL_17964306, EPI_ISL_18682298, EPI_ISL_19125737, EPI_ISL_15753270, EPI_ISL_2582650, EPI_ISL_17964370, EPI_ISL_18682095, EPI_ISL_19125818, EPI_ISL_15753261, EPI_ISL_2582261, EPI_ISL_17964313, EPI_ISL_15753425, EPI_ISL_19125817, EPI_ISL_15753546, EPI_ISL_18143548, EPI_ISL_17964371, EPI_ISL_15753400, EPI_ISL_19125749, EPI_ISL_15753322, EPI_ISL_18143513, EPI_ISL_17964373, EPI_ISL_15753498, EPI_ISL_19125834, EPI_ISL_15753423, EPI_ISL_19048885, EPI_ISL_17964318, EPI_ISL_15753333, EPI_ISL_19125784, EPI_ISL_15753329, EPI_ISL_19048955, EPI_ISL_17964368, EPI_ISL_15753334, EPI_ISL_12544919, EPI_ISL_15753438, EPI_ISL_2583063, EPI_ISL_17964324, EPI_ISL_15753343, EPI_ISL_12544918, EPI_ISL_15753426, EPI_ISL_2595591, EPI_ISL_17964340, EPI_ISL_15753252, EPI_ISL_17964332, EPI_ISL_15753254, EPI_ISL_2595592, EPI_ISL_17964330, EPI_ISL_15753445, EPI_ISL_17964341, EPI_ISL_15753266, EPI_ISL_17964375, EPI_ISL_17964317, EPI_ISL_19048968, EPI_ISL_17964356, EPI_ISL_15753370, EPI_ISL_17964345, EPI_ISL_17964355, EPI_ISL_19048557, EPI_ISL_17964339, EPI_ISL_15753448, EPI_ISL_17964348, EPI_ISL_17964314, EPI_ISL_19048448, EPI_ISL_17964321, EPI_ISL_19048373, EPI_ISL_17964335, EPI_ISL_17964349, EPI_ISL_19048600, EPI_ISL_17964365, EPI_ISL_19048451 |
| RSV subtype A NCBI Accession Numbers  (Figure 4. B) | KJ672432.1, PP376737.1, MH760633.1, MW160782.1, PP376626.1, KJ672434.1, PP376745.1, MH760641.1, MW160785.1, PP376631.1, KJ672441.1, PP376769.1, MH828491.1, MW160787.1, PP376633.1, KJ672442.2, PP376783.1, MH828492.1, MW160795.1, PP376647.1, KJ672447.1, PP376786.1, MH828493.1, MW160801.1, PP376649.1, KJ672467.1, PP376794.1, MH828494.1, MW160803.1, PP376656.1, KJ672478.1, PP376799.1, MH828495.1, MW160819.1, PP376661.1, KM042384.2, PP376800.1, MH828496.1, MW160820.1, PP376662.1, KM042386.1, PP376804.1, MH828497.1, MW160822.1, PP376664.1, KM042392.1, PP376808.1, MH828498.1, MW160824.1, PP376665.1, KU950464.1, PP376814.1, MH828499.1, MW160825.1, PP376666.1, KU950470.1, PP376818.1, MH828500.1, MW160827.1, PP376679.1, KU950472.1, PP376819.1, MH828501.1, MW160828.1, PP376681.1, KU950483.1, PP376821.1, MH828502.1, MW160830.1, PP376690.1, KU950491.1, PP376827.1, MK749890.1, MW582528.1, PP376692.1, KU950518.1, PP376835.1, MK749893.1, MZ515556.1, PP376693.1, KU950519.1, PP376839.1, MK749917.1, MZ515632.1, PP376696.1, KU950520.1, PP377530.1, MN630088.1, MZ515641.1, PP376698.1, KU950523.1, PP377531.1, MN630090.1, MZ515651.1, PP376700.1, KU950531.1, PP377535.1, MN630092.1, MZ515654.1, PP376702.1, KU950544.1, PP377537.1, MN630094.1, MZ515682.1, PP376707.1, KU950590.1, PP377538.1, MN630101.1, MZ515749.1, PP376708.1, KU950623.1, PP377540.1, MN630102.1, MZ515760.1, PP376711.1, KU950628.1, PP377541.1, MN630104.1, MZ515766.1, PP376713.1, KU950629.1, PP377543.1, MN630105.1, MZ515782.1, PP376719.1, KU950638.1, PP377544.1, MN630106.1, MZ515816.1, PP376723.1, KU950666.1, PP377546.1, MT422269.1, MZ515858.1, PP376734.1, KU950683.1, PP377550.1, MT422270.1, MZ515897.1, OQ261746.1, KU950685.1, PP377551.1, MW020596.1, MZ515907.1, OQ595266.1, KU950686.1, PP377553.1, MW020597.1, MZ515910.1, OQ595274.1, KX655626.1, PP377555.1, MW020598.1, MZ515911.1, OQ595279.1, KX655640.1, PP377562.1, MW160746.1, MZ515915.1, OQ595303.1, KX655665.1, PP377570.1, MW160747.1, MZ515969.1, OQ595304.1, KX765886.1, PP377572.1, MW160753.1, MZ515983.1, OQ595306.1, KX765891.1, PP377573.1, MW160758.1, MZ516001.1, OQ595313.1, KX765902.1, PP377575.1, MW160762.1, MZ516040.1, OQ595315.1, KX765904.1, PP377576.1, MW160764.1, MZ516048.1, OQ595316.1, KX765915.1, PP377577.1, MW160768.1, MZ516052.1, OQ595317.1, KX765916.1, PP377578.1, MW160771.1, MZ516053.1, OQ595318.1, KX765917.1, PP377579.1, MW160773.1, MZ516055.1, OQ595319.1, KX765919.1, PP377581.1, MW160774.1, MZ516057.1, OQ595320.1, KX765932.1, PP377582.1, MW160776.1, MZ516058.1, OQ595321.1, KX765939.1, PP377583.1, MW160779.1, MZ516063.1, OQ595322.1, KX765940.1, PP377588.1, MH181903.1, MH182043.1, OQ595328.1, KX765944.1, PP495884.1, MH181986.1, MH182044.1, OQ595330.1, KX765948.1, PX467120, MH181989.1, MH182045.1, OQ595333.1, KX765950.1, PX467124, MH181998.1, MH182048.1, OQ595339.1, KX765951.1, PX467125, MH181999.1, MH182049.1, OQ595355.1, KX765956.1, PX467126, MH182000.1, MH182050.1, OQ595366.1, KX765970.1, PX467127, MH182006.1, MH182054.1, OQ595372.1, KX765972.1, PX467136, MH182008.1, MH182055.1, OQ595373.1, KX765974.1, PX467138, MH182028.1, MH182056.1, OR287841.1, KX765977.1, PX467144, MH182031.1, MH182057.1, OR287862.1, KX894803.1, PX467145, MH182035.1, MH182058.1, OR287863.1, KX894805.1, PX467146, MH182038.1, MH182059.1, OR287865.1, KY654511.1, PP376552.1, MH182039.1, MH182060.1, OR287888.1, KY654513.1, PP376553.1, MH182041.1, MH182061.1, OR287897.1, KY654514.1, PP376556.1, MH182042.1, MH279547.1, OR466354.1, KY654516.1, PP376560.1, MH447960.1, MH290724.1, OR466359.1, KY654517.1, PP376562.1, MH760589.1, MH447953.1, OR666504.1, KY654518.1, PP376566.1, MH760617.1, MH447954.1, OR666505.1, KY883566.2, PP376567.1, MH760621.1, MH447955.1, OR666506.1, KY883567.1, PP376573.1, MH760631.1, MH447956.1, OR666507.1, LC741418.1, PP376574.1, PP376280.1, MH447957.1, OR666508.1, LR699734.1, PP376580.1, PP376281.1, MH447958.1, OR666509.1, LR699737.1, PP376588.1, PP376289.1, MH447959.1, OR666511.1, MF614947.1, PP376590.1, PP376308.1, OR666530.1, OR666512.1, MG773271.1, PP376592.1, PP376309.1, OR666533.1, OR666513.1, MG813984.1, PP376594.1, PP376325.1, OR666534.1, OR666526.1, MG839543.1, PP376612.1, PP376336.1, OR666535.1, OR666527.1, MH181898.1, PP376619.1, PP376346.1, OR666550.1, PP376350.1, OR795340.1, MZ516070.1, PP376374.1, ON237348.1, PP352302.1, OR795341.1, MZ516072.1, PP376382.1, ON237356.1, PP352304.1, OR795342.1, MZ516073.1, PP376394.1, ON237358.1, PP376264.1, OR795343.1, MZ516074.1, PP376395.1, OP320378.1, PP376266.1, OR795344.1, MZ516075.1, PP376402.1, OP320379.1, PP376275.1, OR795345.1, MZ516080.1, PP376405.1, OP320380.1, PP376276.1, OR795346.1, MZ516085.1, PP376407.1, OP320381.1, PP376278.1, OR795347.1, ON237219.1, PP376411.1, OP320382.1, PP376351.1, OR795348.1, ON237223.1, PP376418.1, OP320385.1, PP376352.1, OR795361.1, ON237232.1, PP376424.1, OP320388.1, PP376355.1, OR795362.1, ON237235.1, PP376437.1, OP320389.1, PP376356.1, OR795365.1, ON237237.1, PP376440.1, OP320393.1, PP376364.1, OR795366.1, ON237262.1, PP376445.1, ON237292.1, PP352278.1, OR795367.1, ON237264.1, PP376448.1, ON237294.1, PP352286.1, OR795368.1, ON237265.1, PP376456.1, ON237295.1, PP352287.1, OR795370.1, ON237269.1, PP376466.1, ON237296.1, PP352289.1, OR795371.1, ON237273.1, PP376467.1, ON237297.1, PP352295.1, OR795373.1, ON237278.1, PP376470.1, ON237333.1, PP352296.1, OR795374.1, ON237279.1, PP376473.1, ON237336.1, PP352297.1, OR795380.1, ON237283.1, PP376482.1, ON237341.1, PP352298.1, OY757704.1, ON237289.1, PP376486.1, ON237346.1, PP352301.1, OY757819.1, PP376537.1, PP376504.1, ON237347.1, PP376535.1, PP376549.1, PP376542.1, PP376508.1, PP376546.1, PP376523.1 |

Furthermore, all GISAID genome sequences included in the phylogeny presented in **Figure 4** and associated metadata supporting the findings of this study can be accessed through the persistent digital object identifier <https://doi.org/10.55876/gis8.251205pd>

In addition to the minted DOI, GISAID also communicates the aggregation of GISAID Accession numbers (EPI_ISL_IDs) through the corresponding EPI_SET_251205pd identifier to facilitate both the acknowledgement of all data contributors and the direct retrieval of the underlying data from GISAID used in this study.

**RSV GISAID Data Summary**

| **GISAID Identifier** | **Digital Object Identifier** | **Number of individual viruses** | **Data Collection Range** | **Number of countries/ territories** |
| --- | --- | --- | --- | --- |
| EPI_SET_251205pd | https://doi.org/10.55876/gis8.251205pd | 791 | 2013-01-07 to 2019-12-31 | 33 |

**Table S3.** Samples in which evidence of viral infection was identified. Initial diagnosis refers to the diagnosis assigned by RT-qPCR test upon initial hospital admission. 10 of the 11 positive controls are included here; despite initial diagnosis with alphainfluenza virus infection in one participant, we were not able to recover evidence of infection following mNGS. Read metrics correspond to the metrics obtained through the CZID metagenomic sequencing analysis pipeline to determine evidence of viral infection, including Reads (the number of reads aligning to the identified virus in the NCBI NR/NT database), Length (indicating the average length of the alignment for all reads assigned to the virus), and E-value (the expected value of alignments compared to the NT database). Those that yielded a complete or nearly complete consensus genome sequence are identified by the corresponding NCBI Accession Number. *Note that the Read Metrics column presents metrics from a different analytic pipeline than the one that constructs the consensus genome.

| **BioSample Number** | **Initial Diagnosis** | **Viral Infections** | **Read Metrics** | **Consensus Genome** |
| --- | --- | --- | --- | --- |
| SAMN52841387 | Undiagnosed | Human orthopneumovirus | Reads: 5,370  Length: 1,616,6  E-value: 10e-304 |  |
| SAMN52841388 | Undiagnosed | Human coxsackievirus A | Reads: 36  Length: 187.0  E-value: 10e-89 |  |
| SAMN50934625 | Undiagnosed | Human rhinovirus A | Reads: 493  Length: 7,040.8  E-value: 10e-307 | PX467117 |
| SAMN50934626 | Undiagnosed | Human orthopneumovirus B | Reads: 1,893  Length: 15,287.4  E-value: 10e-307 | PX467118 |
| SAMN52841389 | Undiagnosed | Human coronavirus HKU1 | Reads: 10  Length: 146.0  E-value: 10e-87 |  |
| SAMN52841390 | Undiagnosed | Human coronavirus HKU1 | Reads: 171  Length: 533.9  E-value: 10e-172 |  |
|  |  | Human bocavirus | Reads: 150  Length: 1,126.0  E-value: 10e-212 |  |
| SAMN52841391 | Undiagnosed | Alphainfluenza virus (H3N2) | Reads: 18  Length: 154.5  E-value: 10e-89 |  |
| SAMN52841392 | Undiagnosed | Human mastadenovirus | Reads: 51  Length: 167.8  E-value: 10e-93 |  |
| SAMN52841393 | Undiagnosed | Betanfluenza virus | Reads: 84  Length: 173.6  E-value: 10e-106 |  |
| SAMN52841394 | Undiagnosed | Human metapneumovirus | Reads: 117  Length: 228.6  E-value: 10e-117 |  |
| SAMN52841395 | Undiagnosed | Human rhinovirus A | Reads: 926  Length: 547.2  E-value: 10e-206 |  |
| SAMN52841396 | Undiagnosed | Human orthopneumovirus | Reads: 2,887  Length:14,661  E-value: 10e-302 |  |
| SAMN52841397 | Undiagnosed | Human metapneumovirus | Reads: 52  Length: 146.0  E-value: 10e-86 |  |
| SAMN52841398 | Undiagnosed | Rotavirus A | Reads: 4  Length: 144.5  E-value: 10e-85 |  |
|  |  | Human mastadenovirus C | Reads: 6  Length: 145.0  E-value: 10e-86 |  |
|  |  | Human orthopneumovirus | Reads: 208  Length: 422.0  E-value: 10e-232 |  |
| SAMN50934627 | Undiagnosed | Human rhinovirus C | Reads: 481  Length: 6,518.4  E-value: 10e-305 | PX467119 |
| SAMN50934628 | Undiagnosed | Human coronavirus OC43 | Reads: 914,190  Length: 30,742.2  E-value: 10e-19 | PX467121 |
|  |  | Human orthopneumovirus A | Reads: 69,023  Length: 15,191.4  E-value: 10e-307 | PX467120 |
| SAMN50934629 | Undiagnosed | Enterovirus C99 | Reads: 588  Length: 5,316.8  E-value: 10e-307 |  |
|  |  | Human orthopneumovirus B | Reads: 738  Length: 7,068  E-value: 10e-307 | PX467122 |
| SAMN50934630 | Undiagnosed | Human orthopneumovirus | Reads: 28  Length: 146  E-value: 10e-86 |  |
|  |  | Human mastadenovirus | Reads: 24  Length: 146  E-value: 10e-86 |  |
|  |  | Human rhinovirus A | Reads: 807,103  Length: 7,051.4  E-value: 10e-307 | PX467123 |
| SAMN50934631 | Undiagnosed | Cytomegalovirus | Reads: 30  Length: 147.2  E-value: 10e-83 |  |
|  |  | Human orthopneumovirus A | Reads: 16,707  Length: 15,159.2  E-value: 10e-306 | PX467124 |
| SAMN50934632 | Undiagnosed | Human orthopneumovirus A | Reads: 97,236  Length: 15,199.8  E-value: 10e-307 | PX467125 |
| SAMN50934633 | Undiagnosed | Cytomegalovirus | Reads: 67  Length: 149.3  E-value: 10e-88 |  |
|  |  | Human orthopneumovirus A | Reads:4,237  Length: 15,156,8  E-value: 10e-306 | PX467126 |
| SAMN52841399 | Undiagnosed | Human orthopneumovirus | Reads: 241  Length: 556.7  E-value: 10e-227 |  |
| SAMN50934634 | Undiagnosed | Human orthopneumovirus A | Reads: 4,443  Length: 15,182.1  E-value: 10e-307 | PX467127 |
| SAMN50934635 | Undiagnosed | Human mastadenovirus | Reads: 113  Length: 173.6  E-value: 10e-97 |  |
|  |  | Human rhinovirus A | Reads: 100,000  Length: 6,977.7  E-value: 10e-307 | PX467128 |
| SAMN50934636 | Undiagnosed | Human coronavirus OC43 | Reads: 2,291  Length: 30,527.7  E-value: 10e-306 | PX467129 |
| SAMN52841400 | Undiagnosed | Human orthopneumovirus | Reads: 70  Length: 218.1  E-value: 10e-129 |  |
| SAMN50934637 | Undiagnosed | Human rhinovirus B | Reads: 136,304  Length: 7,212,4  E-value: 10e-307 | PX467130 |
| SAMN50934638 | Undiagnosed | Cytomegalovirus | Reads: 78  Length: 146.0  E-value: 10e-86 |  |
|  |  | Human rhinovirus A | Reads: 23,802  Length: 7,100.7  E-value: 10e-307 | PX467131 |
| SAMN50934639 | Undiagnosed | Human coronavirus NL63 | Reads: 28  Length: 150.9  E-value: 10e-88 |  |
|  |  | Cytomegalovirus | Reads: 32  Length: 146.0  E-value: 10e-86 |  |
|  |  | Betapolyomavirus | Reads: 34  Length: 234.8  E-value: 10e-123 |  |
|  |  | Human mastadenovirus C | Reads: 47  Length: 167.3  E-value: 10e-103 |  |
|  |  | Human bocavirus | Readss: 931  Length: 5,222.9  E-value: 10e-304 | PX467132 |
| SAMN52841401 | Undiagnosed | Human mastadenovirus C | Reads: 12  Length: 144.5  E-value: 10e-86 |  |
| SAMN50934640 | Undiagnosed | Cytomegalovirus | Reads: 4  Length: 144.8  E-value: 10e-86 |  |
|  |  | Human metapneumovirus | Reads: 920  Length: 6,808.0  E-value: 10e-306 | PX467133 |
| SAMN50934641 | Undiagnosed | Human metapneumovirus | Reads: 1,926  Length: 13,327.3  E-value: 10e-307 | PX467134 |
| SAMN52841402 | Undiagnosed | Human alphaherpesvirus 1 | Reads: 2  Length: 146.0  E-value: 10e-87 |  |
|  |  | Human mastadenovirus C | Reads: 2  Length: 146.0  E-value: 10e-86 |  |
| SAMN52841403 | Undiagnosed | Human mastadenovirus C | Reads: 530  Length: 148.3  E-value: 10e-86 |  |
| SAMN52841404 | Undiagnosed | Human metapneumovirus | Reads: 19  Length: 155.8  E-value: 10e-90 |  |
| SAMN50934642 | Undiagnosed | Human rhinovirus C | Reads: 34,901  Length: 6,908.1  E-value: 10e-304 | PX467135 |
| SAMN50934643 | Undiagnosed | Human orthopneumovirus A | Reads: 4,700  Length: 15,199.6  E-value: 10e-307 | PX467136 |
| SAMN50934644 | Undiagnosed | Human mastadenovirus C | Reads: 4  Length: 146.0  E-value: 10e-85 |  |
|  |  | Human rhinovirus A | Reads: 16,725  Length: 7,110.2  E-value: 10e-307 | PX467137 |
| SAMN50934645 | Undiagnosed | Human orthopneumovirus A | Reads: 9,018  Length: 15,146.1  E-value: 10e-306 | PX467138 |
| SAMN50934646 | Undiagnosed | Human rhinovirus C | Reads: 12,641  Length: 7,013.5  E-value: 10e-307 | PX467139 |
| SAMN52841405 | Undiagnosed | Human metapneumovirus | Reads: 8  Length: 161.8  E-value: 10e-90 |  |
| SAMN52841406 | Undiagnosed | Human mastadenovirus | Reads: 28  Length: 145.4  E-value: 10e-86 |  |
|  |  | Human rhinovirus B | Reads: 48  Length: 161.9  E-value: 10e-88 |  |
| SAMN50934647 | Undiagnosed | Human parainfluenzavirus 4 | Reads: 5,032  Length: 16,979.9  E-value: 10e-307 | PX467140 |
| SAMN50934648 | Undiagnosed | Human rhinovirus C | Reads:350  Length: 1,984.2  E-value: 1-e-307 | PX467141 |
| SAMN52841407 | Undiagnosed | Human orthopneumovirus | Reads: 183  Length: 404.0  E-value: 10e-196 |  |
| SAMN50934649 | Undiagnosed | Human rhinovirus C | Reads: 841  Length: 6,896.0  E-value: 10e-306 | PX467142 |
| SAMN52841408 | Undiagnosed | Human rhinovirus A | Reads: 46  Length: 435.5  E-value: 10e-172 |  |
|  |  | Cytomegalovirus | Reads: 40  Length: 138.1  E-value: 10e-81 |  |
| SAMN52841409 | Undiagnosed | Human rhinovirus A | Reads: 24  Length: 199.4  E-value: 10e-124 |  |
| SAMN52841410 | Undiagnosed | Human rhinovirus A | Reads: 370  Length: 3,143.5  E-value: 10e-306 |  |
| SAMN52841411 | Undiagnosed | Human metapneumovirus | Reads: 424  Length: 2,438.2  E-value: 10e-281 |  |
| SAMN52841412 | Undiagnosed | Human mastadenovirus C | Reads: 28  Length: 146.1  E-value: 10e-86 |  |
| SAMN52841413 | Undiagnosed | Human rhinovirus C | Reads: 12  Length: 146.0  E-value: 10e-76 |  |
| SAMN52841414 | Undiagnosed | Human rhinovirus A | Reads:460  Length:6,854.6  E-value:10e-306 |  |
| SAMN52841415 | Undiagnosed | Human mastadenovirus C | Reads: 100  Length: 145.5  E-value: 10e-86 |  |
| SAMN52841416 | Undiagnosed | Human coronavirus HKU1 | Reads: 190  Length: 243.0  E-value: 10e-136 |  |
| SAMN52841417 | Undiagnosed | Human parainfluenzavirus 1 | Reads:30  Length: 145.8  E-value: 10e-83 |  |
| SAMN50934650 | Undiagnosed | Human metapneumovirus | Reads: 4,952  Length: 6,610.1  E-value: 10e-307 | PX467143 |
| SAMN52841418 | Human orthopneumovirus | Human orthopneumovirus | Reads: 81  Length: 185.5  E-value: 10e-100 |  |
|  | Human rhinovirus |  |  |  |
| SAMN52841419 | Human orthopneumovirus | Human orthopneumovirus | Reads: 42  Length: 165.6  E-value: 10e-94 |  |
| SAMN52841420 | Human orthopneumovirus | Human orthopneumovirus | Reads: 38  Length: 156.9  E-value: 10e-90 |  |
| SAMN50934651 | Human orthopneumovirus | Human orthopneumovirus A | Reads: 6,267  Length: 14,954.9  E-value: 10e-304 | PX467144 |
| SAMN50934652 | Human orthopneumovirus | Cytomegalovirus | Reads: 26  Length: 146.0  E-value: 10e-87 |  |
|  |  | Human orthopneumovirus A | Reads: 75,662  Length: 15,133.9  E-value: 10e-306 | PX467145 |
| SAMN52841421 | Human orthopneumovirus | Cytomegalovirus | Reads: 90  Length: 146.0  E-value: 10e-86 |  |
|  |  | Human orthopneumovirus | Reads: 3,088  Length: 7,511.6  E-value: 10e-303 |  |
| SAMN50934653 | Human orthopneumovirus | Human orthopneumovirus A | Reads: 17,166  Length: 15,073.6  E-value: 10e-306 | PX467146 |
| SAMN52841422 | Human orthopneumovirus | Human orthopneumovirus | Reads: 325  Length: 424.7  E-value: 10e-184 |  |
| SAMN52841423 | H3N2 | Alphainfluenza(H3N2) virus | Reads: 1,273  Length: 1,786.6  E-value: 10e-305 |  |

**Figure S1**. Nasopharyngeal swabs had on average >80% quality control scores, compression ratios (a ratio of host to non-host reads; lower values indicate more non-host reads present in a sample) close to 1, and a high number of non-host reads remaining after filtering and post-sequencing processing (top). Reads were filtered for size and complexity and to remove reads corresponding to human hosts (bottom). Overall, after iterative filtering, nasopharyngeal swabs had sufficient quality metrics to proceed to analysis.


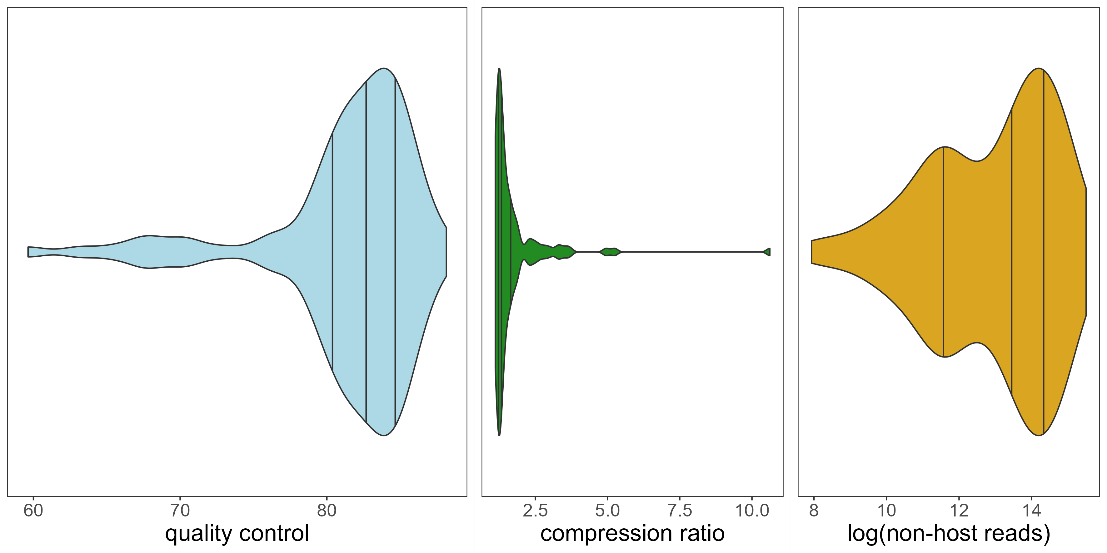


**
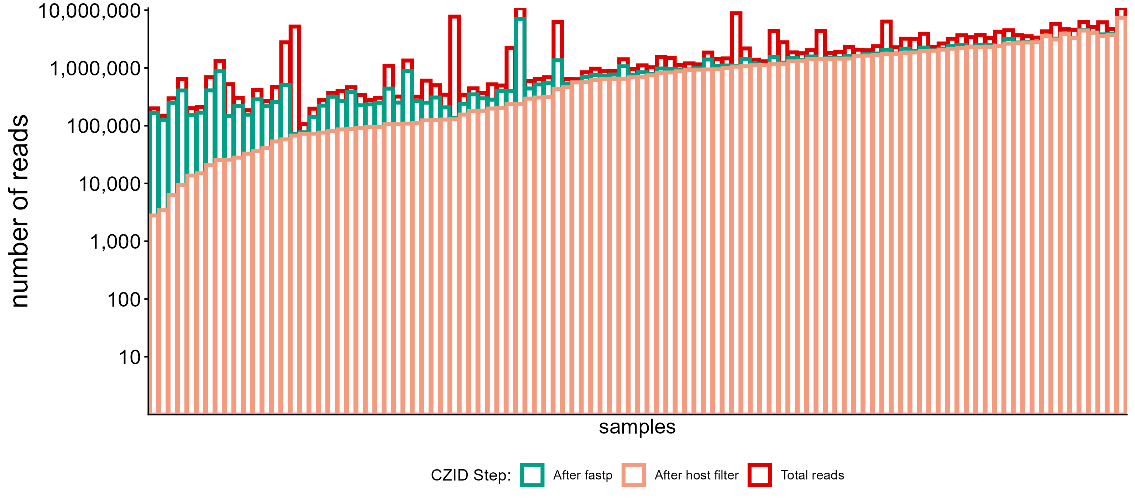
Figure S2.** Alignment plots of multiplex PCR primers with consensus genomes. Primer alignment was conducted using previously published primers^7^ in Geneious Prime and visualized using the package ‘ggmsa’ in R 4.4.1.


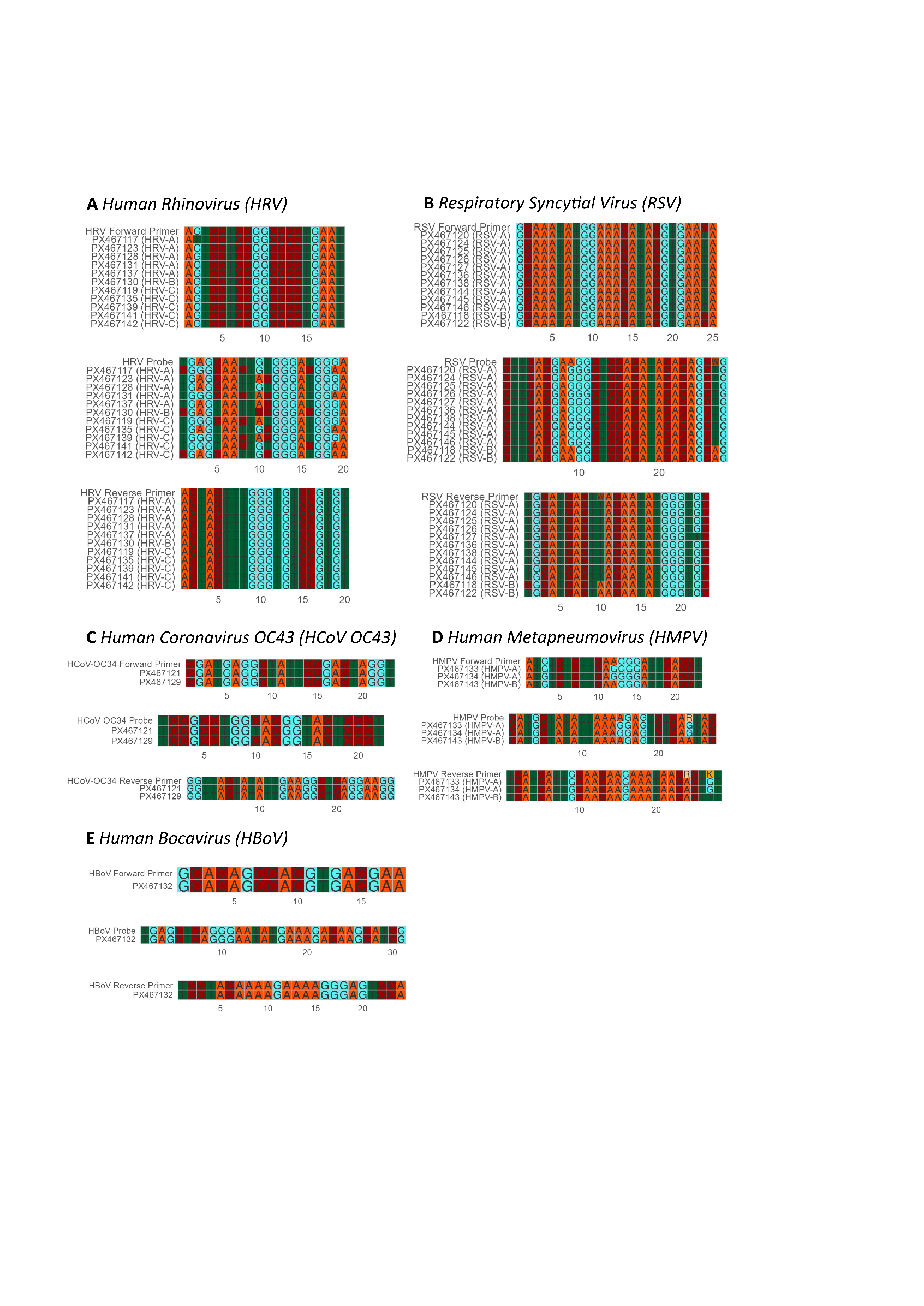

Supplement: Supplementary file 1 — Supplementary Material 1 [file 12879_2026_13715_MOESM1_ESM.docx]
